# Striated preferentially expressed gene deficiency leads to mitochondrial dysfunction in developing cardiomyocytes

**DOI:** 10.1007/s00395-023-01029-7

**Published:** 2023-12-26

**Authors:** Gu Li, He Huang, Yanshuang Wu, Chang Shu, Narae Hwang, Qifei Li, Rose Zhao, Hilaire C. Lam, William M. Oldham, Souheil EI-Chemaly, Pankaj B. Agrawal, Jie Tian, Xiaoli Liu, Mark A. Perrella

**Affiliations:** 1 Division of Newborn Medicine, Department of Pediatrics, Brigham and Women’s Hospital, Boston, MA 02115 USA; 2 Department of Cardiology, and Department of Pulmonary, Children’s Hospital, Chongqing Medical University, Chongqing, 400015 China; 3 Department of Anesthesiology, The Second Affiliated Hospital, Chongqing Medical University, Chongqing, 400010 China; 4 Division of Genetics and Genomics, The Manton Center for Orphan Disease Research, Boston Children’s Hospital, Boston, MA 02115 USA; 5 Division of Neonatology, Department of Pediatrics and Jackson Health System, University of Miami Miller School of Medicine, Miami, FL 33136 USA; 6 Division of Pulmonary and Critical Care Medicine, Department of Medicine, Brigham and Women’s Hospital, 75 Francis Street, Boston, MA 02115 USA

**Keywords:** *Speg*, Dilated cardiomyopathy, Mitochondria, PGC-1α phosphorylation

## Abstract

A deficiency of striated preferentially expressed gene (*Speg*), a member of the myosin light chain kinase family, results in abnormal myofibril structure and function of immature cardiomyocytes (CMs), corresponding with a dilated cardiomyopathy, heart failure and perinatal death. Mitochondrial development plays a role in cardiomyocyte maturation. Therefore, this study investigated whether *Speg* deficiency ( – / – ) in CMs would result in mitochondrial abnormalities. *Speg* wild-type and *Speg*^−/−^ C57BL/6 littermate mice were utilized for assessment of mitochondrial structure by transmission electron and confocal microscopies. Speg was expressed in the first and second heart fields at embryonic (*E*) day 7.5, prior to the expression of mitochondrial Na^+^/Ca2^+^/Li^+^ exchanger (NCLX) at E8.5. Decreases in NCLX expression (E11.5) and the mitochondrial-to-nuclear DNA ratio (E13.5) were observed in *Speg*^−/−^ hearts. Imaging of E18.5 *Speg*^−/−^ hearts revealed abnormal mitochondrial cristae, corresponding with decreased ATP production in cells fed glucose or palmitate, increased levels of mitochondrial superoxide and depolarization of mitochondrial membrane potential. Interestingly, phosphorylated (*p*) PGC-1α, a key mediator of mitochondrial development, was significantly reduced in *Speg*^−/−^ hearts during screening for targeted genes. Besides Z-line expression, Speg partially co-localized with PGC-1α in the sarcomeric region and was found in the same complex by co-immunoprecipitation. Overexpression of a Speg internal serine/threonine kinase domain in *Speg*^−/−^ CMs promoted translocation of pPGC-1α into the nucleus, and restored ATP production that was abolished by siRNA-mediated silencing of PGC-1α. Our results demonstrate a critical role of Speg in mitochondrial development and energy metabolism in CMs, mediated in part by phosphorylation of PGC-1α.

**Supplementary Information:**

The online version contains supplementary material available at 10.1007/s00395-023-01029-7.

## Introduction

Dilated cardiomyopathy is one of the most common heart diseases and causes of death in both newborns and adults [14], and death due to abnormal sarcomeric proteins is not uncommon [20, 34]. The myosin light chain kinase (MLCK) family of proteins is known to be important in phosphorylating sarcomeric proteins [21] and gene mutations in this family leads to the development of dilated cardiomyopathy in humans and mice [46]. Striated preferentially expressed gene (*Speg*), also known as striated muscle preferentially expressed protein kinase, a member of the MLCK family, shares homology with other family members and contains two tandemly arranged serine/threonine kinase domains (SK) [30]. The *Speg* gene locus contains four different isoforms, and the Spegα and Spegβ isoforms are expressed in striated muscle (cardiac and skeletal) and co-localize with cardiac troponin T/I in cardiomyocytes (CMs) [29, 30]. We and our colleagues have demonstrated that haploinsufficiency of *Speg* in adult mice, under pathological conditions such as pressure overload, leads to an increased risk of cardiac hypertrophy and subsequent decompensated heart failure [37, 44]. Recently, additional investigations have shown that Speg regulates calcium homeostasis by phosphorylating sarcoplasmic reticulum (SR) Ca2^+^ ATPase (SERCA) 2a and ryanodine receptor (RyR) 2 on SR [6, 7, 24, 26, 36], thereby playing a role in the contractility of myofibers. The importance of Speg goes beyond animal studies, as more than 20 human patients to date have been identified with recessive *Speg* mutations, and cardiac dysfunction was seen in the majority of these patients [1, 32].

During murine development, the disruption of *Speg* causes a dilated cardiomyopathy, corresponding with ventricular dysfunction and death in the perinatal period with no mice surviving beyond a few hours after birth [29, 30]. *Speg* deficient ( – / – ) neonates have abnormal cardiac contractility, with thin and disorganized myofibrils of the CMs, and reduced phosphorylation of sarcomeric proteins such as tropomyosin [30]. Mitochondrial development has been shown to play a role in cardiomyocyte maturation [13, 17], and interestingly mitochondrial diseases have been reported to be associated with a high mortality rate at birth [10, 11]. There is a high density of mitochondria in cardiac muscle, with the highest ATP consumption per unit tissue weight in the body, but energy reserves in the heart are relatively limited [40]. Thus, right after birth mitochondria must adapt to the transition from low levels of oxygen in utero to a more aerobic environment with the associated rapid increase in energy demand. Oxidative phosphorylation, which occurs in mitochondria, is the most efficient way to generate ATP [3, 9, 16, 28, 40]. Mitochondrial density and mass accordingly increase sharply during the perinatal period [5, 9, 31]. All of these processes rely on normal mitochondrial maturation at birth. Therefore, we hypothesize that Speg deficiency leads to abnormalities in mitochondrial structure and function, which drives ventricular dysfunction and perinatal mortality.

The peroxisome proliferator-activated receptor γ coactivator (PGC) 1 family is central to the regulatory network of mitochondrial development and function in CMs [9, 25, 40]. Expression of PGC-1α, a member of this family, is regulated by extracellular and physiological signals [25]. For example, the expression of PGC-1α is repressed in numerous models of heart failure, which implicates PGC-1α as an important factor in the maladaptive energetic profile of failing hearts [40]. During cardiac development, PGC-1α expression is gradually increased over time, rises rapidly before birth and peaks on neonatal day 1 [9]. The PGC-1α protein translocates into the nucleus, interacting with transcription factors to promote expression of nuclear genes encoding mitochondrial proteins (NUGEMPs). Specifically, PGC-1α is known to associate with nuclear respiratory factor (NRF) 1/2, estrogen-related receptor (ERR), and peroxisome proliferator-activated receptor (PPAR) that are involved in mitochondrial biogenesis and maturation, as well as regulating energy metabolism, production of reactive oxygen species (ROS), and cell death [9, 40, 47]. Posttranslational modifications of the PGC-1α protein plays key roles in its expression, localization, and activity [33]. Phosphorylation, acetylation, methylation, and ubiquitination are predominantly responsible for the transcriptional activity and stability of PGC-1α [33]. Interestingly, the activity of PGC-1α is increased when phosphorylated by a number of factors, including p38 mitogen-activated protein kinase (MAPK), adenosine monophosphate activated protein kinase (AMPK), c-AMP-response element binding protein (CREB), calcium/calmodulin-dependent protein kinase (CaMK), and glycogen synthase kinase 3β (GSK3β) [9, 33, 40, 47]. To our knowledge, it remains to be elucidated whether MLCK family proteins, such as Speg, phosphorylate PGC-1α. This study will investigate the impact of Speg on cardiac mitochondrial development, maturation, and function. Furthermore, we will explore whether these effects of Speg are achieved by modulating the posttranslational phosphorylation of PGC-1α.

## Methods

### Animals

*Speg*^−/−^ mice were previously generated as described [30]. In brief, exons 8, 9, and 10 of Speg were deleted in the genetically modified mice. A Speg targeting vector was generated by first cloning a 5’ fragment containing intron 6 and exon 7 into pBluescript vector (Stratagene), followed by a 3’ fragment containing part of intron 10 and exons 11–14. A fragment of lacZ was then inserted between the 5’ and 3’ fragments of the targeting vector. A PGK-neo cassette was subsequently subcloned 3’ of lacZ for positive selection. A thymidine kinase cassette (PGK-TK) was ligated at the 5’-end to allow negative selection with gancyclovir. Genotyping was performed by PCR (Supplementary Fig. 1. Primers detailed in Supplementary Tab.1). Since the *Speg*^−/−^ mice die shortly after birth, all studies were performed on the offspring of *Speg* heterozygous ( ±) breeding. Thus, *Speg*^+/+^ mice were littermates of *Speg*^−/−^ mice. The use of mice and the studies performed were carried out in accordance with the Public Health Service policy on the humane care and use of laboratory animals, and the protocol was approved by the Institutional Animal Care and Use Committee of Brigham and Women’s Hospital. Euthanasia methods of mice used in the experiments followed the AVMA Guidelines for the Euthanasia of Animals. Euthanasia for mouse embryos and altricial neonates was performed by hypothermia using an ice slurry (with the animals not coming in direct contact with the ice), and following loss of movement decapitation using sharp surgical scissors. In adult mice, euthanasia was performed by administering anesthesia (ketamine and xylazine), followed by removal of the hearts.

### Tissue and cell staining

#### β-Galactosidase activity

Embryos were stained for β-galactosidase activity as previously described [30]. Briefly, embryos were washed with cold PBS + 2 mM MgCl_2_ and fixed in 0.2% glutaraldehyde PBS + 2 mM MgCl_2_ for 30–60 min depending on the age of the embryos. After washing in 0.02% NP-40, the embryos were stained with 1 mg/mL of 5-bromo-4-chloro-3-indolyl-β-D-galactopyranoside in 0.1 M phosphate buffer including 20 mM Tris, 2 mM MgCl_2,_ 0.01% Na deoxycholate, 0.02% NP-40, 5 mM potassium ferricyanide, and 5 mM potassium ferrocyanide, at pH 7.3. Staining was done for 30–60 min depending on the age of embryos, at 37 °C.

#### Immunofluorescent staining

Hearts were arrested in diastole by cadmium chloride (0.1 M) perfusion and fixed in 10% formalin and embedded in paraffin. Sections (5 μM) were microwaved in 10 mM citrate buffer (pH 6.0) for 10 min to retrieve antigens and then blocked with 10% donkey serum for 30 min. CMs were fixed with 2% paraformaldehyde for 15 min, permeabilized with 0.1% Triton for 10 min, and blocked with 2% BSA for 1 h at room temperature[29, 30]. The heart sections or CMs were incubated with primary antibodies (Supplementary Tab.2) at 4 °C overnight, following by secondary antibodies conjugated with fluorescence at 37 °C for 1 h and nuclei staining with 4′,6-diamidino-2-phenylindole (DAPI) at 37 °C for 10 min**.** Images were analyzed using fluorescence or confocal microscopy[29, 30].

### Isolation of neonatal CMs

CMs were isolated using the neonatal CMs kit (Supplementary Tab.3) following the manufacturer’s instruction. Briefly, hearts were harvested from the neonates and tissues were dissociated in digestion buffer (D2 + EC) at 37 °C. The supernatants were collected every 12 min, and this process was repeated six to seven times until all tissues were digested. The cells were collected by spinning down the supernatants and pre-plated for 45 min at 37 °C to reduce the contamination of fibroblasts. The unattached cells were seeded onto precoated plates and cultured in neonatal CM culture medium with serum.

### Mitochondrial assays

#### ATP content

E18.5 CMs were isolated (see details in “Isolation of neonatal CMs”) and cultured on 96-well plates with 25,000 cells per well. The attached cells were lysed, and intracellular ATP concentration was measured by the ATPlite assay system (Supplementary Tab.3) on a luminescent plate reader, according to the manufacturer’s specifications.

#### Real-time ATP

In brief, E18.5 CMs were isolated, pooled (*n* = 3–4 hearts), and seeded in laminin-coated wells of an XF96 plate at 20,000 cells/well, in 100μL neonatal CM culture medium for 48 h at 37 °C in a humidified atmosphere of 95% air and 5% CO_2_. Before the assay, the medium was replaced by 180μL of bicarbonate-free Seahorse assay medium, pH 7.4, supplemented with a final concentration of 10 mM glucose, 1 mM pyruvate, and 2 mM glutamine, and CMs were pre-incubated for 1 h at 37 °C in CO_2_-free incubator. Oxygen consumption rate (OCR) and extracellular acidification rate (ECAR) were measured using a Seahorse extracellular flux analyzer. After three basal measurements, the assay compounds oligomycin and rotenone/antimycin A at final concentrations of 2μM and 0.5/0.5 μM, respectively, were sequentially injected. OCR and ECAR data were collected and used to calculate the ATP production rate. Detailed reagents are provided in Supplementary Tab.3.

#### Palmitate oxidation stress

CMs were seeded as above and cultured in neonatal CM culture medium for 48 h, followed by changing to 100μL L-carnitine DMEM medium with 1% serum, 0.5 mM L-carnitine, 0.5 mM glucose (low level), 1 mM glutamine and 1% penicillin–streptomycin overnight. Fatty acid oxidation (FAO) assay medium was freshly prepared in DMEM with 0.5 mM L-carnitine and 1 mM glucose. CMs were cultured in FAO assay medium in a CO_2_-free incubator for 1 h. Before running a Seahorse extracellular flux analyzer, BSA or palmitate–BSA (Pal–BSA, 30μL of 1 mM) was added to the corresponding wells. Measurements were obtained at baseline, and following injections with oligomycin (Oligo, 2 μM), trifluoromethoxy carbonylcyanide phenylhydrazone, carbonyl cyanide 4-(trifluoromethoxy) phenylhydrazone (2 μM), and rotenone/antimycin A (0.5/0.5 μM)[2]. Data were normalized to cell counts after staining with Hoechst 33,342 (1μg/mL) and then imaging using a PerkinElmer Operetta high-content, wide-field, fluorescence imaging system. ImageJ was then performed to count the cell number. Detailed reagents are provided in Supplementary Tab.3.

#### Mitochondrial ROS

CMs were labeled with 5μΜ MitoSox™ Red (Supplementary Tab.3) at 37 °C for 10 min and then washed. The cells were resuspended for flow cytometry.

#### Mitochondrial membrane potential assay

CMs were labeled with tetramethylrhodamine, methyl ester (TMRM, Supplementary Tab.3) at a final concentration of 20nΜ TMRM in culture medium, at 37 °C for 30 min. The cells were treated with 50μΜ carbonyl cyanide m-chlorophenyl hydrazone (CCCP) for 5 min at 37 °C to serve as a positive control. Flow cytometry assessment was subsequently performed.

#### Transmission electron microscopy (TEM)

Hearts from E18.5 embryos were harvested as previously described [29, 30]. Briefly, hearts were fixed in 0.1 M sodium cacodylate buffer containing 2% PFA and 2.5% glutaraldehyde for 2 h at room temperature and then overnight at 4 °C. The hearts were then postfixed in 1% osmium tetroxide/1.5% potassium ferrocyanide and dehydrated, followed by embedding in Epon/Araldite resin. The hearts were sectioned, and TEM images were taken. Mitochondrial number and size (area) were analyzed using ImageJ and represented as mitochondria per image area (µm^2^). Length and width of the cristae lumen were measured as the distance from the top to the junction of cristae, and the distance at the middle level of cristae, respectively. Mitochondria were classified into categories I, II, III and IV, based on their morphology, and the presence and number of tubular cristae that extend and connect to the mitochondrial periphery [17, 48]. Class I are very immature mitochondria that have rare or no cristae and an expanded matrix. Class II are immature mitochondria that have sparse, tubular cristae with few tubular cristae connections to the periphery and a slightly expanded matrix. Class III are almost mature mitochondria that have better defined cristae and many tubular cristae connections to the periphery and a mostly compacted matrix with only occasional lucencies. Class IV are fully mature mitochondria that have abundant, organized/laminar cristae with multiple tubular connections to the periphery and a compacted matrix with no lucencies [17, 48].

#### Airyscan microscopy

CMs were isolated from E18.5 *Speg*^+*/*+^ and *Speg*^*−/−*^ hearts and cultured in neonatal CM culture medium for 3 days. Live CMs were stained with nonyl acridine orange (NAO, Supplementary Tab. 3) for 30 min and scanned by a Zeiss LSM880 with AiryScan FAST Confocal microscope [43]. The color threshold of the Fiji imaging processing package was performed to select the outlines of mitochondrial inner membrane and cristae for calculating their areas.

### Quantitative real-time PCR (qRT-PCR)

Total RNA was extracted using TRIzol reagent and cDNA was generated using SuperScript III First-Strand Synthesis System [29, 30]. To quantify the amount mtDNA present per nuclear genome, total DNA was extracted from hearts using lysis buffer containing 100 mM NaCl,10 nM EDTA, 0.5% SDS, 20 mM Tris–HCl, proteinase K and RNase A[38]. qRT-PCR was performed using SYBR Green kit. Mouse primers are provided in Supplementary Tab.1.

### Western blotting

Hearts or 293 T cells were homogenized in RIPA buffer, containing protease inhibitor and phosphatase inhibitor [29, 30]. Protein 40μg was separated in 7.5% or 4–20% Tris–glycine–SDS-polyacrylamide gels and transferred onto PVDF membranes. The membranes were blocked with 5% non-fat milk in TBST and incubated with primary antibodies (Supplementary Tab.2), following HRP-conjugated secondary antibodies.

#### Protein co-immunoprecipitation (IP)

Heart tissue was homogenized with Pierce™ IP lysis buffer, containing inhibitors of protease and phosphatase. 1500μg of protein was pre-cleared with 30μL of magnetic Protein-A Dynabeads for 1 h at 4ºC. The supernatant was incubated with a primary antibody of either Speg or PGC-1α overnight on a rotator at 4 °C and then incubated with 50μL of magnetic Protein-A Dynabeads for 2 h at 4 °C. The bead–Ab–Ag complex was washed with lysis buffer and then extracted with Laemmli sample buffer. Western blotting was then performed on the immunoprecipitated lysates. A detailed list of the antibodies is shown in Supplementary Tab.2.

### In vitro transfection

#### Transfection of the construct expressing the internal serine/threonine kinase (SK) domain of Speg

The Speg SK expression plasmid was created as described [18]. Briefly, a portion of Speg cDNA containing the internal SK domain (amino acid residues 1598–1862) was amplified by RT-PCR using the following two primers, *Speg* F4935 5’-ggatccCGAGGAAGAAGACTTAGCGAC-3’ (the ggatcc is an added BamHI site and does not present in the cDNA) and *Speg* R5729 5’-TTCTGTTTTGAACCAAGGATG-3’. This cDNA fragment was ligated to an amino-terminal FLAG sequence in the plasmid vector pCMV-FLAG-2 to make the pFLAG-CMV-2-Speg SK construct.

CMs or 293 T cells were transfected with the SK plasmid or vector (Vec) using Fugene HD[18]. Briefly, CMs or 293 T cells were plated onto 12-well plates for 48 h and then treated with 1 mL culture medium containing 0.5μg DNA and 1μL Fugene HD for 48 h. The cells were harvested for ATP assays, immunofluorescent staining, or Western blotting.

#### Silencing of PGC-1α

CMs were transfected with a negative control (Neg, a non-targeting siRNA) or PGC-1α siRNA Oligo Duplex (Ppargc-1α siRNAs, OriGene #SR427524), according to the manufacturer’s protocol. In brief, CMs were cultured on 24-well plates. PGC-1α siRNAs (equally mixed with 3 siRNAs) were diluted in Opti-MEM Medium and Lipofectamine RNAiMAX Reagent (100/3 in v/v) at a final concentration of 10 nM and incubated for 15 min. CMs were treated with 100µL mixed solution in 900µL culture medium, containing BrdU to inhibit fibroblast growth, for 36 h. Following transfection with Vec or SK plasmid, the medium was removed, and fresh culture medium placed on the cells for 8 h.

### Statistical analysis

One- or two-way ANOVA was performed depending on the number of experimental factors. For comparisons between two groups, Student’s unpaired *t* test was used. Statistical significance was accepted at *P* < 0.05. For further details, see the figure legends.

## Results

### Speg expression prior to mitochondrial detection in early fetal hearts

**Fig. 1:**
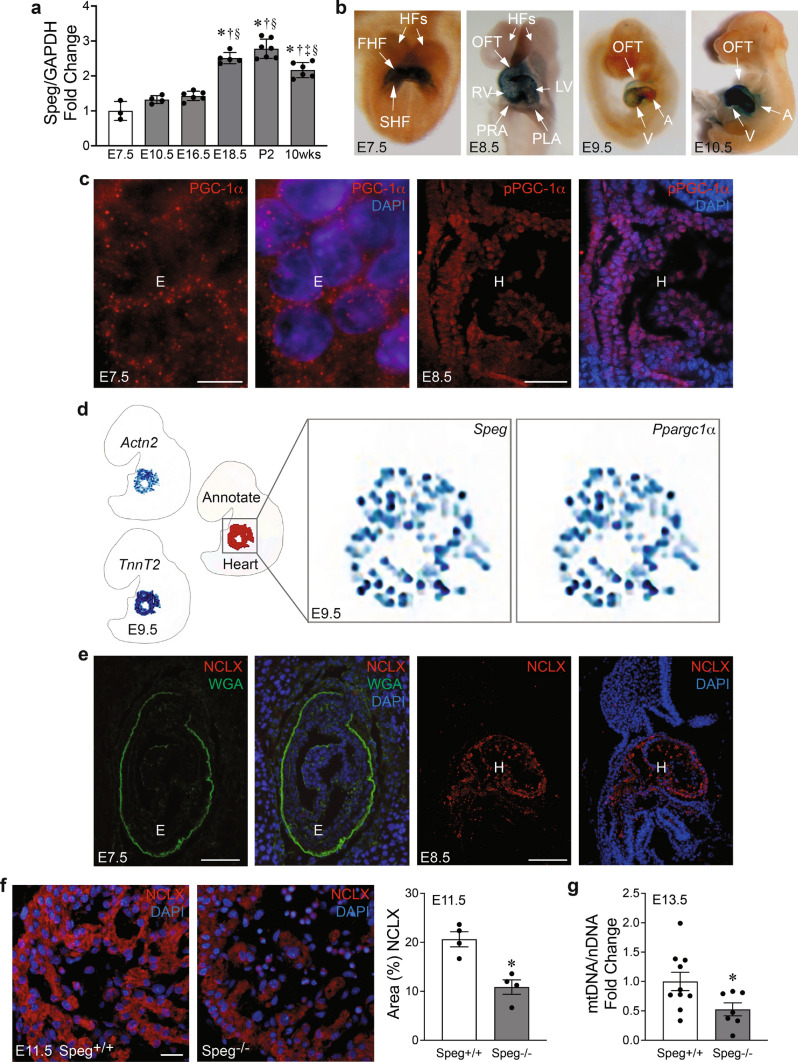
Characterization of *Speg* and mitochondrial gene expression in early developing mouse hearts. Embryos (*E*) or hearts (*H*) of different gestational, postnatal (*P*) and weeks (wks) of age after birth were harvested. **a** qRT-PCR for *Speg* expression in embryos (white bar) and hearts (gray bars) of mice, *n* = 3–7 for each time point. **b** Representative images of embryos staining for β-galactosidase (β-gal, blue). *FHF* first heart field; *SHF* second heart field; *HFs* head folds; *OFT* outflow tract, *RV *right ventricle, *LV *left ventricle, *PRA *pulmonary right artery, *PLA *pulmonary left artery, *V *ventricle, *A *atrium. Representative images of immunofluorescent staining for **c** peroxisome proliferator-activated receptor g coactivator 1α (PGC-1α, red) or phospho (*p*) PGC-1α (red). **d** Transcript levels of *Actn2, TnnT2, Speg*, and *Ppargc1a* from C57BL/6 mouse embryo hearts at E9.5 extracted from a publicly available spatial transcriptomic data set [8]. **e** Na^+^/Ca^2+^/Li^+^ exchanger (NCLX, red) and DAPI (blue, nuclei) of embryos and hearts. Wheat germ agglutinin (WGA) conjugated with Alexa Fluor 488 staining for embryo border (green). **f** Representative confocal images of immunofluorescent staining for NCLX and DAPI at E11.5 *Speg*^+/+^ and *Speg*^−/−^ hearts. Quantitation of the NCLX staining is shown as a % area of staining in bar graph, *n* = 4 embryos for each group. **g** qRT-PCR of the amount mitochondrial DNA (mtDNA) present per nuclear genome DNA (nDNA) of E13.5 *Speg*^+/+^ and *Speg*^−/−^ hearts, *n* = 4 hearts for each group. The data in all bar graphs are presented as mean ± SEM. One-way ANOVA was performed in **a**, *p* < 0.0001 with significant comparisons * versus E7.5 embryos, † versus E10.5 hearts, ‡ versus E16.5 hearts, and § versus P2 hearts. Unpaired *t* tests were performed in **f** and **g**. * *p* ≤ 0.0386 versus *Speg*^+/+^ hearts. Scale bars represent 5μm for theleft two panels and 50μm for the right two panels in **c**, 100μm in e and 25μm in **f**

We have previously shown that Speg was expressed in CMs at embryonic (E) day 11.5 [30]. The heart is the first organ to form at E7.5; thus, this study further investigated Speg expression at early gestational ages, up until the age of young adult mice. We harvested embryos at E7.5, and hearts from E10.5 through 10-week-old and performed qRT-PCR. GAPDH mRNA levels showed no notable changes during development (Supplementary Fig. 2, left) and were used to normalize *Speg* gene expression. Speg mRNA was detected in E7.5 fetuses, and expression in hearts significantly increased by E18.5 and remained elevated in young adult hearts (Fig. 1a, Supplementary Fig. 2, right). When we previously generated Speg^−/−^ mice, the gene-targeting strategy effectively knocked in the bacterial lacZ reporter gene under the transcriptional control of the *Speg*α promoter [30]. We thus assessed lacZ expression by staining for β-galactosidase activity in genetically modified embryos to determine the location of *Speg*α promoter activity during development. β-galactosidase stained positively in the first and second heart fields at E7.5 and in the hearts of E8.5–E10.5 fetuses (Fig. 1b). We previously demonstrated that β-galactosidase staining was positive only in CMs, but negative in smooth muscle cells of coronary arteries, and the cardiac outflow tract was not detected until E18.5 [30].

Due to the importance of mitochondrial maturation and biogenesis in the maturation of CMs, and a key role of PGC-1α in this process, we next characterized expression of PGC-1α. Immunofluorescence staining revealed that PGC-1α and phosphorylated (*p*) PGC-1α were positive in E7.5 embryos and E8.5 hearts, respectively (Fig. 1c). A publicly available spatial transcriptomic data set (MOSTA, Mouse Organogenesis Spatiotemporal Transcriptomic Atlas) was analyzed in the sagittal section of mouse embryos at E9.5, and we localized CMs using the specific markers *Actinin alpha 2* (*Actn2*) and *cardiac troponin T2* (*TnnT2*) [8]. Expression of *Speg* and PGC-1α (*Ppargc1a*) displayed an analogous temporal and spatial expression pattern in E9.5 CMs (Fig. 1d). To further understand the emergence of mitochondria in the developing murine heart, immunofluorescence staining was performed for the mitochondrial markers Na^+^/Ca^2+^/Li^+^ exchanger (NCLX) and translocase of the outer mitochondrial membrane complex subunit 20 (TOM20). These data revealed that NCLX was not detectable in the heart until E8.5 (Fig. 1e). TOM20 was also expressed at E8.5 in the heart and throughout the embryo (Supplementary Fig. 3). However, embryos showed no expression of either protein at E7.5. Importantly, immunofluorescence staining for SERCA2α and caveolin-3 revealed that SR and t-tubules, respectively, were undetectable in E9.5 hearts (Supplementary Fig. 4), implicating the presence of mitochondria earlier than SR and t-tubules. Meanwhile, *Speg*^*−/−*^ hearts revealed less pPGC-1α expression in nuclei compared with *Speg*^+*/*+^ hearts at E9.5 (Supplementary Fig. 5). Furthermore, immunofluorescence staining of E11.5 fetuses showed less expression of NCLX in *Speg*^−/−^ hearts compared with *Speg*^+/+^ mice (Fig. 1f). qRT-PCR analysis further confirmed that mitochondrial (mt) DNA abundance was also significantly reduced in *Speg*^−/−^ compared with *Speg*^+/+^ hearts at E13.5 (Fig. 1g). Taken together, these data suggest that *Speg* is expressed in fetal hearts as early as E7.5, the gestational age at which the heart begins to develop, while markers of mitochondria are not yet detected. Moreover, mitochondrial abundance is less in *Speg*^−/−^ embryonic hearts.

### Abnormal mitochondrial cristae in the hearts of *Speg*^−/−^ mice

**Fig. 2:**
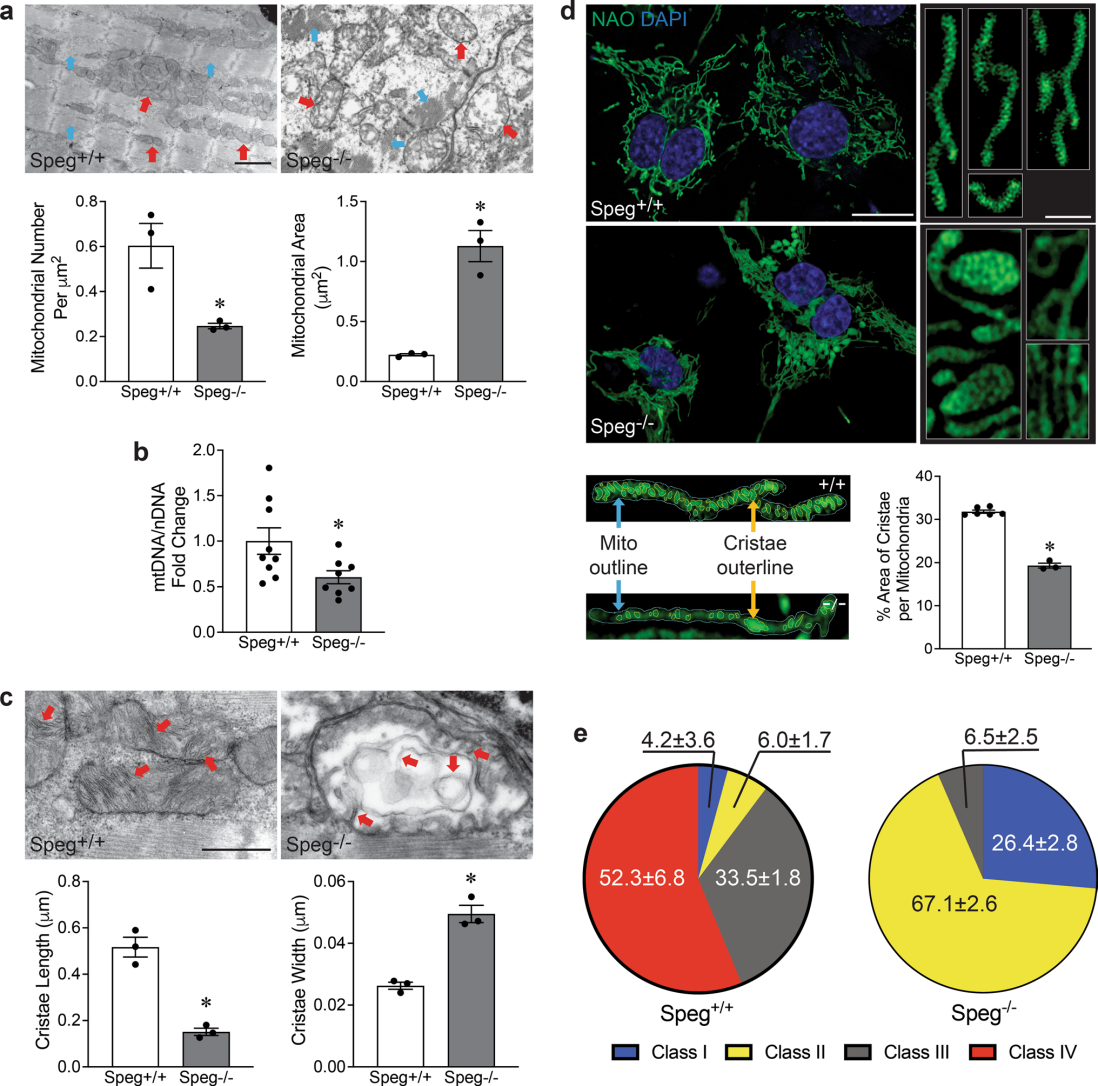
Abnormalities of mitochondrial structure in E18.5 *Speg*^−/−^ hearts. **a** Representative TEM images. Quantitation of mitochondrial number per µm^2^ and mitochondrial area (µm^2^) from TEM images in the bar graphs, *n* = 3 hearts for each group and three images for each mouse. Red and blue arrows point to mitochondria and myofibrils, respectively. **b** qRT-PCR of the amount mitochondrial DNA (mtDNA) present per nuclear genome DNA (nDNA), *n* = 8–9 hearts for each group. **c** Representative TEM images of mitochondrial cristae. Quantitation of length and width of mitochondrial cristae in bar graph, *n* = 3 hearts for each group and 150–300 mitochondria for each group. Red arrows point to mitochondrial cristae. **d** Representative AiryScan images of NAO staining for mitochondrial inner membrane and cristae in lower (left) and higher (right) magnifications. The lower images represent the outlines of cristae (yellow line) and mitochondrial (blue line), areas selected by the Fiji color threshold. Quantitation of % area cristae per mitochondria in bar graph, CMs from three to six E18.5 hearts for each group and 50 mitochondria for each mouse, and total 531–1077 cristae. **e** Pie graph shows the distribution of mitochondrial class I (blue), II (yellow), III (gray) and IV (red), *n* = 3 hearts for each group and three images for each mouse. The data are presented as mean ± SEM, with t-tests performed in all bar graphs. * *p* ≤ 0.0336 versus *Speg*^+/+^ hearts. Scale bars represent 1μm in **a**, 500 nm in **c**, 20μm for left and 2μm for right panels in **d**

We investigated the ultrastructure of E18.5 hearts by TEM. The mitochondria of *Speg*^+/+^ hearts were distributed along myofibers and filled with abundant tubular cristae and a compact matrix (Fig. 2a**,** left), whereas *Speg*^−/−^ hearts had large, dilated, and disorganized mitochondria occupied by abnormal cristae and a translucent matrix (Fig. 2a, right). Quantitative analysis of TEM images revealed that *Speg*^−/−^ hearts have decreased mitochondrial numbers and increased size compared with *Speg*^+/+^ hearts (Fig. 2a, bar graphs). qRT-PCR quantitation further confirmed that the ratio of mtDNA to nuclear (n) DNA was significantly reduced in *Speg*^−/−^ compared with *Speg*^+/+^ CMs (Fig. 2b).

Mitochondrial cristae are folds in the inner membrane that provide a large amount of surface area for chemical reactions and ATP synthesis. TEM image analysis revealed shorter and wider mitochondrial cristae in *Speg*^−/−^ compared with *Speg*^+/+^ hearts (Fig. 2c). Airyscan microscopy was performed on live CMs stained with NAO, which specifically labels lipids of the inner mitochondrial membrane, including cristae. Abnormalities of mitochondria and cristae morphology were shown in *Speg*^−/−^ CMs (Fig. 2d**,** images), Furthermore, the area of cristae per mitochondria was measured by the Fiji imaging processing package, as illustrated in the schematic images of Fig. 2d bottom. The percent area of cristae in mitochondria was significantly decreased in *Speg*^*−/−*^ compared to *Speg*^+*/*+^ CMs (Fig. 2d, bar graph). This abnormal morphology of mitochondria led us to investigate whether the membrane channels were abnormal. Mitochondrial permeability transition pore (mPTP) was assessed using calcein/cobalt quenching and showed that calcein AM fluorescent intensity in *Speg*^*−/−*^ CMs was the same as in *Speg*^+*/*+^ CMs, suggesting an altered opening of mPTP was not an issue in E18.5 *Speg*^*−/−*^ CMs (Supplementary Fig. 6a). Furthermore, labeling of Fluo-4 AM, fluorescent calcium indicator, showed that there were no differences in mitochondrial calcium concentrations between E18.5 *Speg*^+*/*+^ and *Speg*^*−/−*^ CMs (Supplementary Fig. 6b). Immunoprecipitating Speg from adult heart protein lysates yielded bands ~ 250 kDa, corresponding to Spegα (Supplementary Fig. 6c, top panels). In the Speg pulldown complex, blotting with voltage-dependent anion-selective channel (VDAC), the mitochondrial calcium uniporter (MCU), and NCLX antibodies showed that these proteins are undetectable in this Speg complex (Supplementary Fig. 6c, bottom panels).

Mitochondria fall into four classes during maturation, including class I (very immature), class II (immature), class III (almost mature), and class IV (mature) [48]. We found that the major mitochondrial subpopulations in E18.5 *Speg*^+/+^ hearts were class III and class IV, 33.5 ± 1.8 and 52.3 ± 6.8%, respectively. In contrast, the predominant subtypes of mitochondria in *Speg*^−/−^ heart were class I and II, 26.4 ± 2.5% and 67.1 ± 2.6%, respectively (Fig. 2e). MitoTracker staining showed that mitochondria were mainly distributed in the perinuclear region of *Speg*^−/−^ CMs (consistent with immaturity [17]) and scattered in the cytoplasm of more mature *Speg*^+/+^ CMs (Supplementary Fig. 7).

### Mitochondrial functional defects of *Speg*^−/−^ CMs

**Fig. 3:**
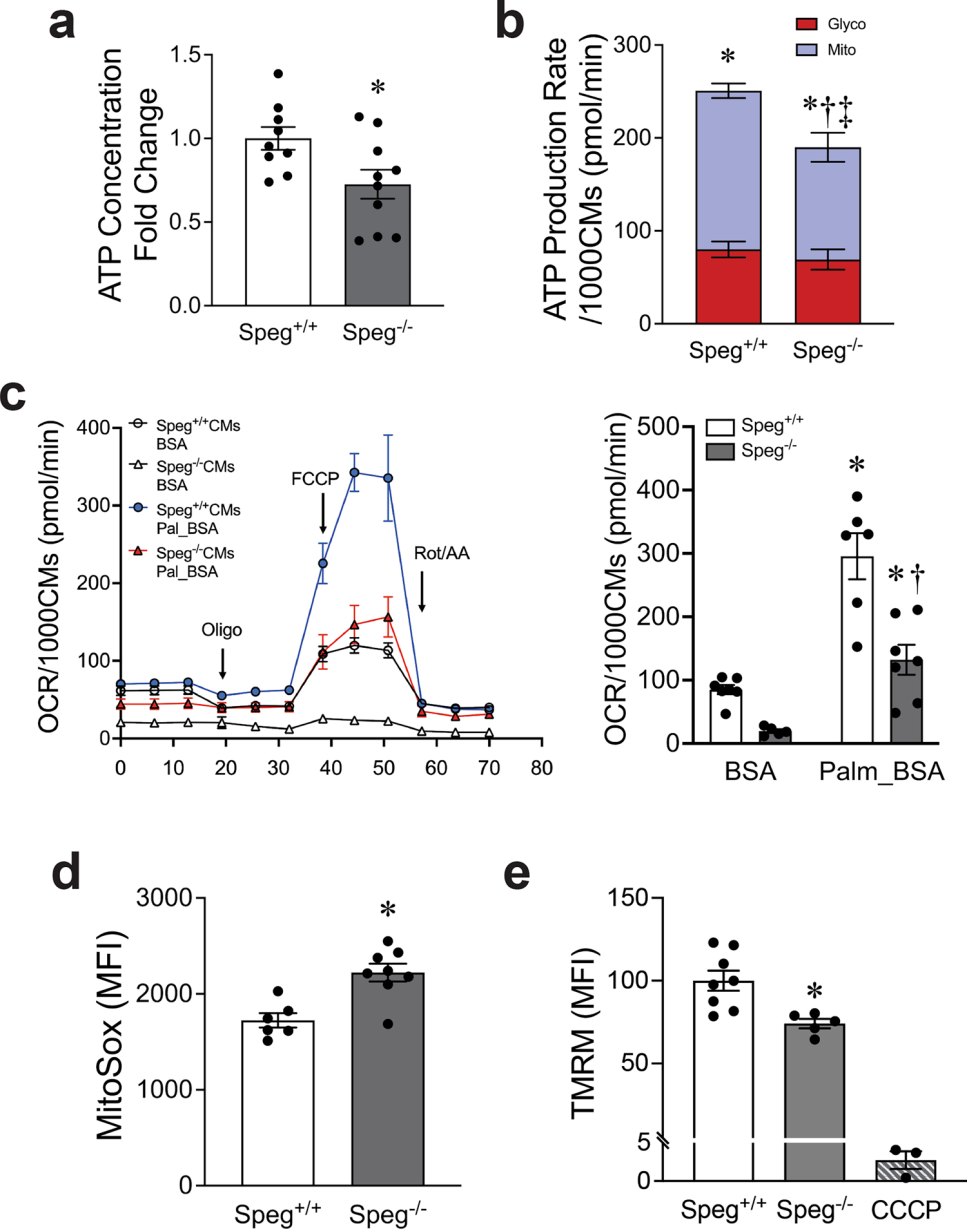
Abnormalities of mitochondrial function in E18.5 *Speg*^−/−^ hearts. **a** Quantification of ATP concentration of CMs, from *n* = 9–10 hearts for each group. **b** Quantification of ATP production rate per 1000 CMs by Seahorse XF real-time ATP rate assay. Glyco glycolytic ATP (red bars), Mito mitochondrial ATP (blue bars). Total ATP = GlycoATP + MitoATP, *n* = 5 hearts for each group. **c** Palmitate FAO substrate assay. OCR curves (left) and quantification (right) of Max OCR per 1000 *Speg*^+*/*+^ and *Speg*^−/−^ CMs in BSA, Palm_BSA (palmitate conjugated with BSA), *n* = 5–7 from pooled hearts for each group. Quantification of flow cytometry assay for **d** MitoSox in MFI, *n* = 6–8 hearts for each group, and **e** TMRM in MFI, *n* = 5–8 hearts for each group, using CCCP as a positive control for disruption of membrane potential. The data are presented as mean ± SEM. t-tests were performed in **a, d** and **e**, comparing *Speg*^+/+^ with *Speg*^−/−^ groups. * *p* =  ≤ 0.0257 versus *Speg*^+/+^ CMs or hearts. Two-way ANOVA was performed in **b**. *p < 0.0001, mitoATP versus glycoATP, †*p* = 0.007, mitoATP in *Speg*^*−/−*^ versus *Speg*^+*/*+^ CMs*.* ‡*p* = 0.0410, total ATP in *Speg*^*−/−*^ versus *Speg*^+*/*+^ CMs. Two-way ANOVA was performed in **c**. *p ≤ 0.0123, versus their corresponding BSA CMs, †*p* < 0.0002 versus Palm_BSA *Speg*^+*/*+^ CMs

CMs are among the cells with the most abundant mitochondria, which are essential for cardiac function. To further understand whether abnormal cristae morphology is associated with mitochondrial dysfunction, we first measured the ATP content of CMs. The ATP concentration was significantly lower in *Speg*^−/−^ compared with *Speg*^+/+^ CMs (Fig. 3a). In addition, a Seahorse assay was performed to assess ATP production rate. *Speg*^−/−^ CMs showed a decrease of both total and mitochondrial ATP production rate compared with *Speg*^+/+^, while ATP production from glycolysis was the same in the two groups (Fig. 3b). Fatty acid β-oxidation is important for CMs development and function in neonates [22]. We next performed a FAO stress assay. Maximum OCR was reduced in *Speg*^−/−^ compared with *Speg*^+/+^ CMs when using palmitate, a long-chain fatty acid, as substrate (Fig. 3c). Taken together, these data suggest that ATP concentration was primarily decreased due to a reduction in the ATP production rate in *Speg*^*−/−*^ CMs. Thus, we will perform an assessment of ATP concentration as a reflection of ATP production in the remaining experiments. Flow cytometry was also performed to assess MitoSox for mitochondrial superoxide, and TMRM for mitochondrial membrane potential. Data revealed increased reactive oxygen species and a decrease in membrane potential in the *Speg*^−/−^ compared with the *Speg*^+/+^ CMs (Fig. 3d and e, respectively). JC-1 labeling confirmed a depolarization in *Speg*^−/−^ CMs (Supplementary Fig. 8). Western blotting did not detect cleaved-PARP or cleaved caspase 3 in *Speg*^−/−^ hearts (Supplementary Fig. 9a). Furthermore, terminal deoxynucleotidyl transferase dUTP nick end labeling (TUNEL) staining confirmed no increase in TUNEL-positive cells in *Speg*^−/−^ compared with *Speg*^+/+^ hearts (Supplementary Fig. 9b). Taken together, we found no evidence of increased apoptosis in *Speg*^−/−^ hearts.

### Down-regulation of the PGC-1α pathway in *Speg*^*−/−*^ hearts

**Fig. 4:**
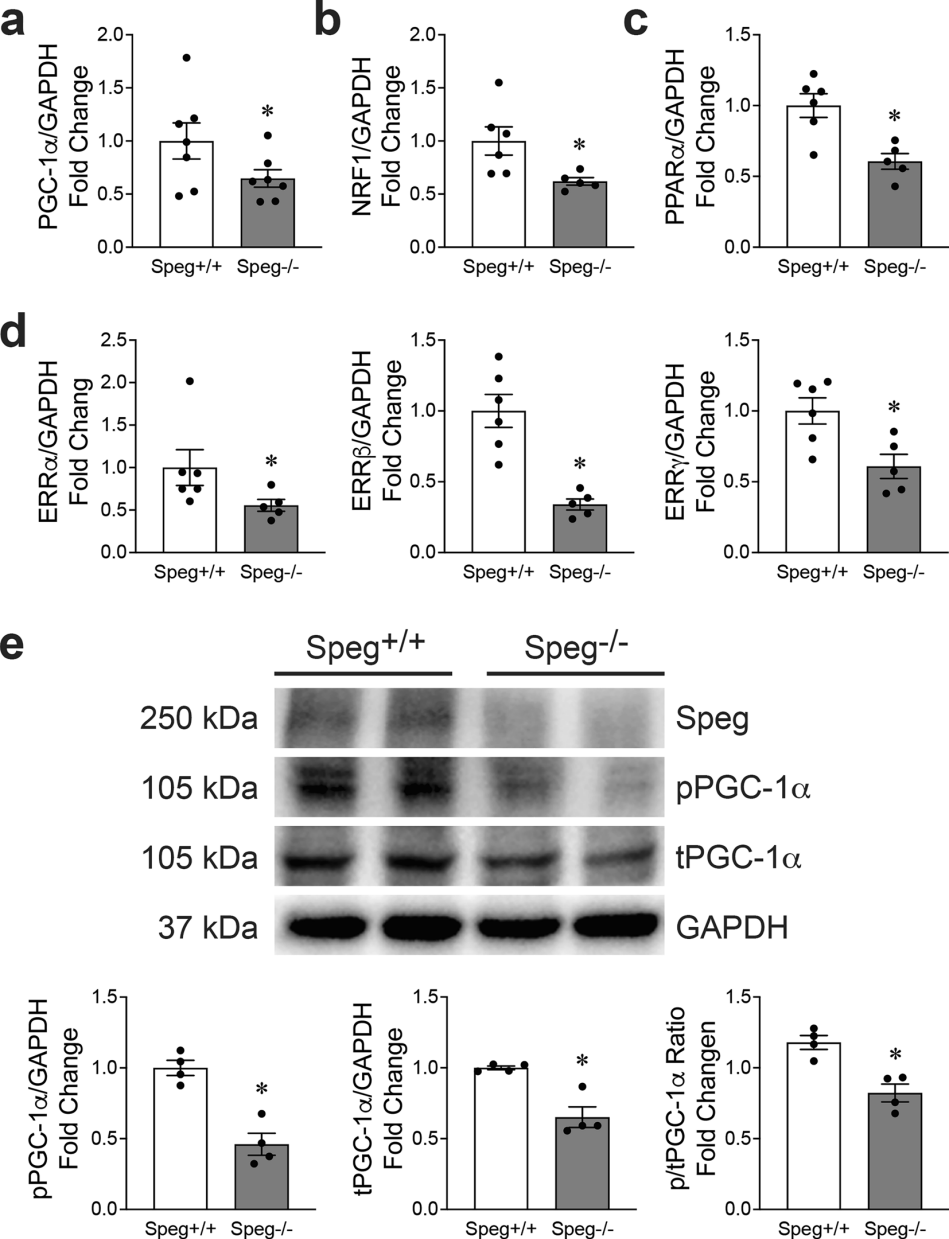
Regulation of the mitochondrial biogenesis pathway by Speg. qRT-PCR for **a** PGC-1α, **b** NRF1, **c** PPARα, and **d** ERR α, β and γ, normalized by GAPDH and shown as fold change to *Speg*^+/+^ hearts, *n* = 5–7 hearts for each group. **e** Representative Western blots for Speg (first row), phosphorylated (p, second row) and total (t, third row) PGC-1α, and GAPDH (fourth row). Quantitation of Western blotting in the bar graphs for pPGC-1α, tPGC-1α and the ratio of p/t PGC-1α, *n* = 4 hearts for each group. The data in all bar graphs are shown as mean ± SEM, with *t*-test comparisons. * *p* ≤ 0.0428 versus *Speg*^+/+^ group

PGC-1α is a key regulator of mitochondrial function and biogenesis during development [9, 25, 40, 47]. qRT-PCR showed that PGC-1α was significantly decreased in *Speg*^−/−^ compared with *Speg*^+/+^ hearts (Fig. 4a). In addition, downstream genes of PGC-1, including NRF1, PPARα, and ERRα, ERRβ and ERRγ (Fig. 4b–d) were also significantly downregulated in *Speg*^−/−^ compared with *Speg*^+/+^ hearts. There was also a trend for a reduction in PGC-1β, NRF2, PPARβ/α and PPARγ, but this difference was not statistically significant (Supplementary Fig. 10). Protein levels of total, phosphorylated, and the ratio of phosphorylated to total PGC-1α were decreased in *Speg*^−/−^ compared with *Speg*^+/+^ hearts (Fig. 4e).

### Formation of a Speg and PGC-1α complex

**Fig. 5:**
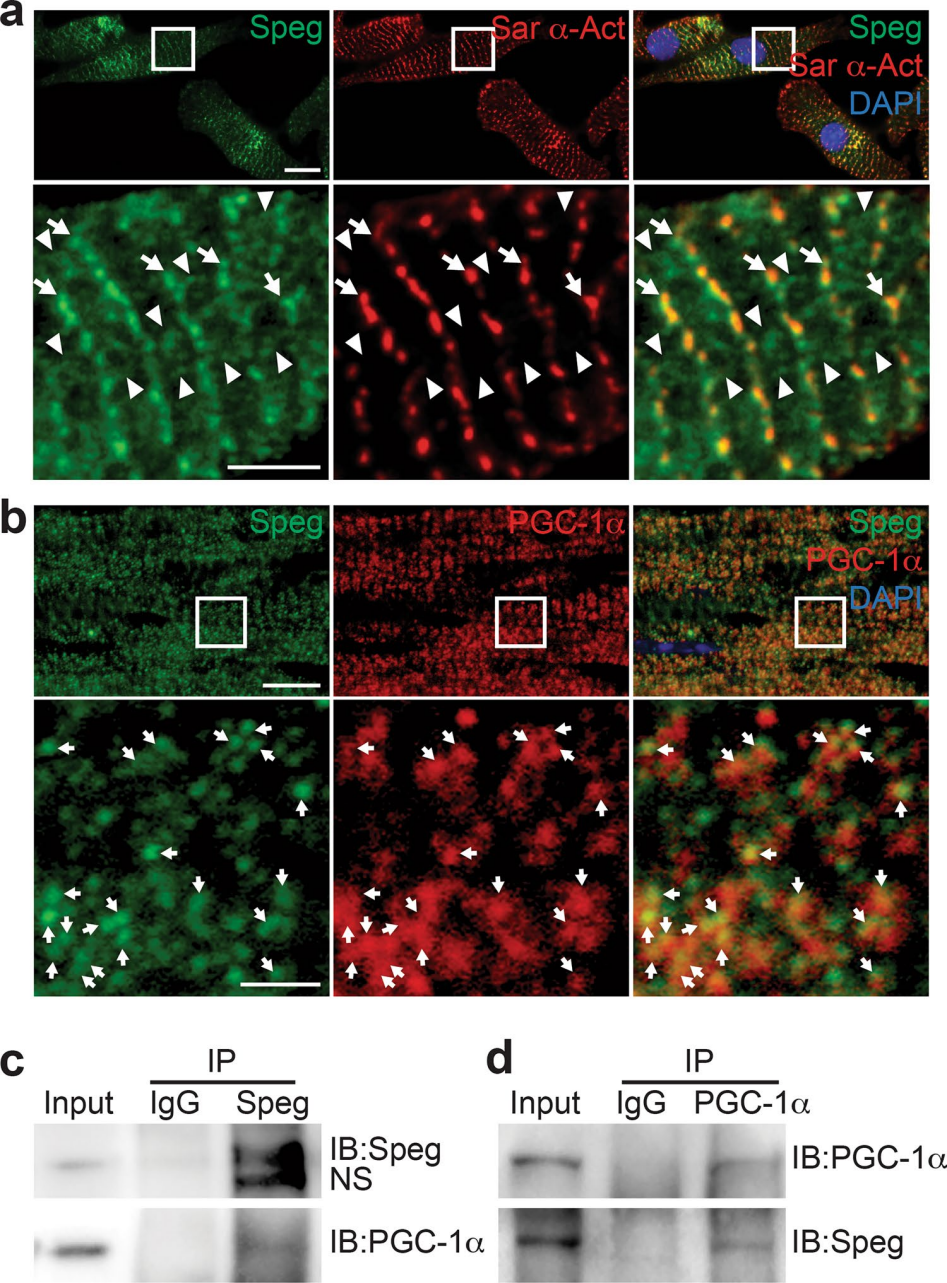
Complex of Speg and PGC-1α. **a** Representative images of immunofluorescent co-staining for Speg (green), Sar α-actinin (Sar α-Act, red) and DAPI (blue, nuclei) in neonatal CMs. The higher magnification images, from the area of their corresponding white boxes, are shown in the bottom panels. Arrows point to positive co-staining for Speg and Sar α-actinin. Arrow heads point to positive staining for Speg. **b** Representative images of immunofluorescent co-staining for Speg (green), PGC-1 α(red) and DAPI (blue) in adult mouse hearts. The higher magnification images, from the area of their corresponding white boxes, are shown in the bottom panels. Arrows point to positive co-staining for Speg and PGC-1 α. Scale bars represents 10μm for the top panels, and 2μm for the bottom panels in **a**, and 20μm for the top panels, and 2μm for the bottom panels in **b**. Total protein was extracted from *Speg*^+/+^ adult hearts.** c** Representative blots of total protein immunoprecipitated without antibody (input), with normal rabbit IgG (IgG), and with rabbit anti-mouse Speg antibody (Speg). The membrane was immunoblotted with anti-Speg (IB: Speg, top row) and anti-PGC-1α antibodies (IB: PGC-1α, bottom row). ns: nonspecific band. **d** Representative of total protein immunoprecipitated without antibody (input), with normal rabbit IgG, and with rabbit anti-mouse PGC-1α antibody (PGC-1α). The membrane was immunoblotted with anti-PGC-1α (IB: PGC-1α, top row) and Speg (IB: Speg, bottom row)

To elucidate how Speg interacts with PGC-1α, we next investigated established activators of PGC-1α, using Western blotting. pCaMKII/CaMKII, pMAPK/MAPK, pAKT/AKT, pGSK3β/GSK3β, pPKA/PKA, SIRT1, AMPKα1/2 and CaN were not different in *Speg*^−/−^ compared with *Speg*^+/+^ hearts (Supplementary Fig. 11). Immunofluorescent staining of CMs showed co-localization of Speg and sarcomeric α-actinin at the z-line site. Speg alone was also found in the area between z-lines, distributed in a branched network structure (Fig. 5a), which partially overlapped with PGC-1α (Fig. 5b). To confirm co-localization of Speg and PGC-1α, the yellow spots (merged green and red) in the area between Z-lines were selected by color threshold and measured using the color histogram of the Fiji imaging processing package. The scatter plots of red and green pixel intensities were consistent with a positive linear association of Speg and PGC-1α (Supplementary Fig. 12). To further confirm an interaction of Speg and PGC-1α, total protein was extracted from adult hearts and subjected to IP using a Speg antibody. Western blotting was performed on the precipitated proteins and immunoblotting with antibodies targeting Speg and PGC-1α revealed bands of ~ 250 kDa and ~ 105 kDa, corresponding to Spegα and PGC-1α, respectively (Fig. 5c). A complementary IP experiment using a PGC-1α antibody, followed by Western blotting with antibodies targeting PCG-1α and Speg, revealed bands of ~ 105 kDa and ~ 250 kDa, corresponding to PGC-1α and Speg, respectively (Fig. 5d).

### Rescue of mitochondrial function in *Speg*^*−/−*^ CMs by expressing the Speg SK domain to promote phosphorylation of PGC-1α

**Fig. 6:**
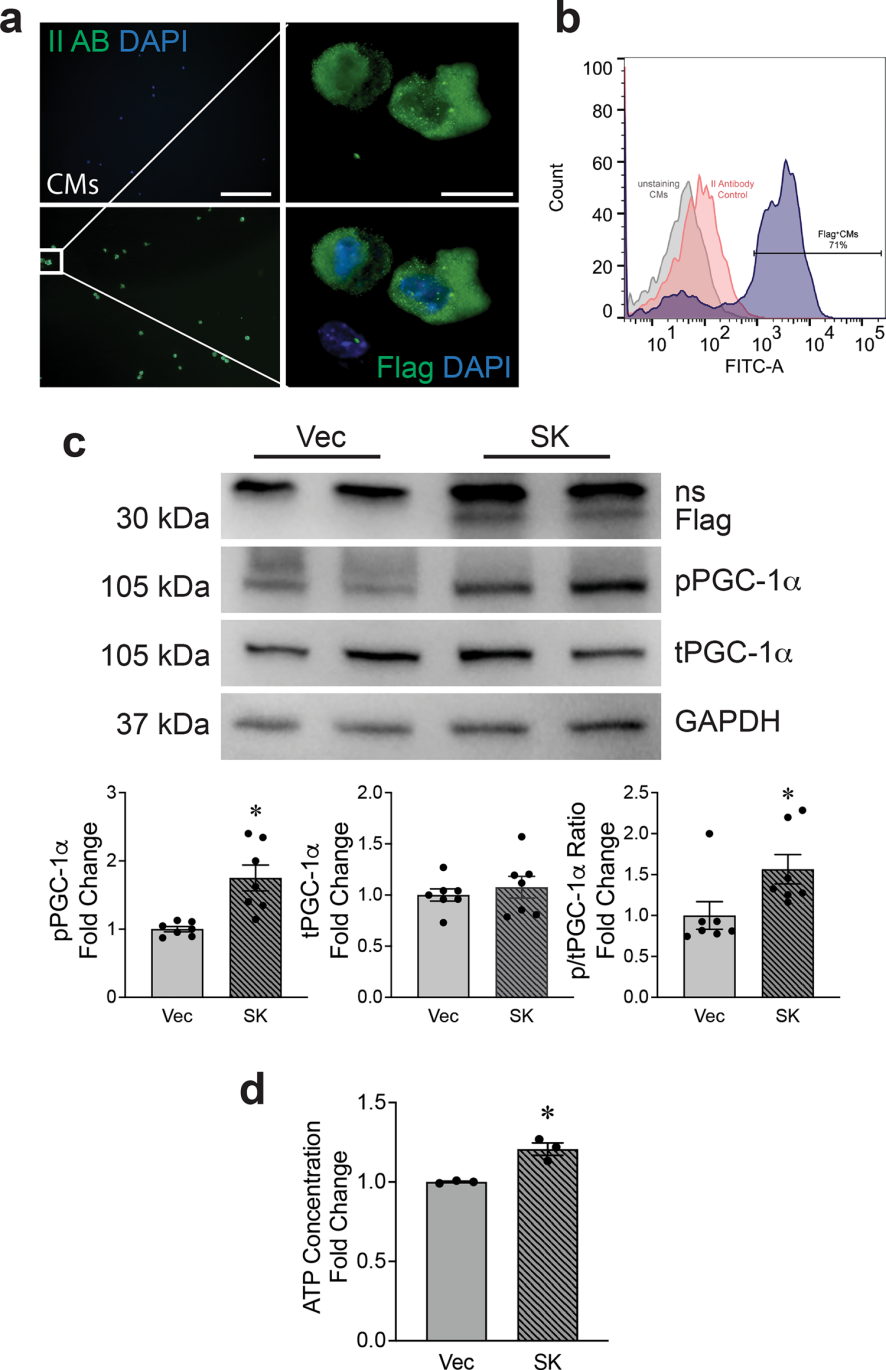
Phosphorylation of PGC-1α by Speg SK. The plasmid DNA constructs containing either pFLAG-CMV-2 vector (Vec) or Speg SK were transfected into CMs and 293 T cells. **a** and **b** CMs were staining for Flag M2. Representative images of immunofluorescent staining (**a**) and flowcytometry histogram (**b**). **c** The total protein was extracted from transfected 239 T cells. Representative Western blots using anti-Flag (top row), phosphorylated (p) peroxisome proliferator-activated receptor g coactivator 1α (PGC-1α, second row), total (t)PGC-1α (third row) and GAPDH (fourth row) antibodies are shown. Ns nonspecific bands. Quantitation of Western blotting in bottom bar graphs for tPGC-1α, pPGC-1α and ratio of p to t PGC-1α, *n* = 7–8 independent experiments for each group. **d** Quantitation of ATP concentration in 293 T cells transfected with Vec or Speg SK, *n* = 3 independent experiments for each group. The data in bar graphs are presented as mean ± SEM. t-tests were performed comparing 293 T cells transfected with Vec and SK. * *p* ≤ 0.0111 versus Vec group

Posttranslational modifications of PGC-1α, including phosphorylation, play a key role in regulating its activity and translocation into the nucleus to target downstream mitochondria associated genes [47]. To address whether Speg, a member of the MLCK family, plays a role in this process [30], we generated an SK construct, containing an internal SK, and cloned this SK into pCMV-Flag2 [18]. Transfection efficiency and Flag-targeted SK (Flag-SK) expression were assessed. CMs transfected with this plasmid were stained using a Flag M2 antibody. Immunofluorescent imaging and flow cytometry showed Flag-positive cells were ~ 78 ± 11% and ~ 71% (Fig. 6a and b, respectively). Due to the limitation of neonatal CMs, *Speg* non-expressing 293 T cells were used to confirm the expression of the Flag-SK and PGC-1α by Western blotting. Data showed an ~ 30 kDa band, corresponding to the Flag-SK (Fig. 6c, top blot). pPGC-1α and the ratio of phosphorylated to total PGC-1α protein were significantly increased in 293 T cells transfected with Speg SK compared with cells transfected with vector (Vec) control (Fig. 6c, left and right bar graphs), while PGC-1α total protein was not altered in 293 T cells transfected with Speg SK (Fig. 6c, middle bar graph). Transfection of 293 T cells with Speg SK demonstrated a significant increased ATP concentration compared with Vec control-transfected cells (Fig. 6d).

**Fig. 7:**
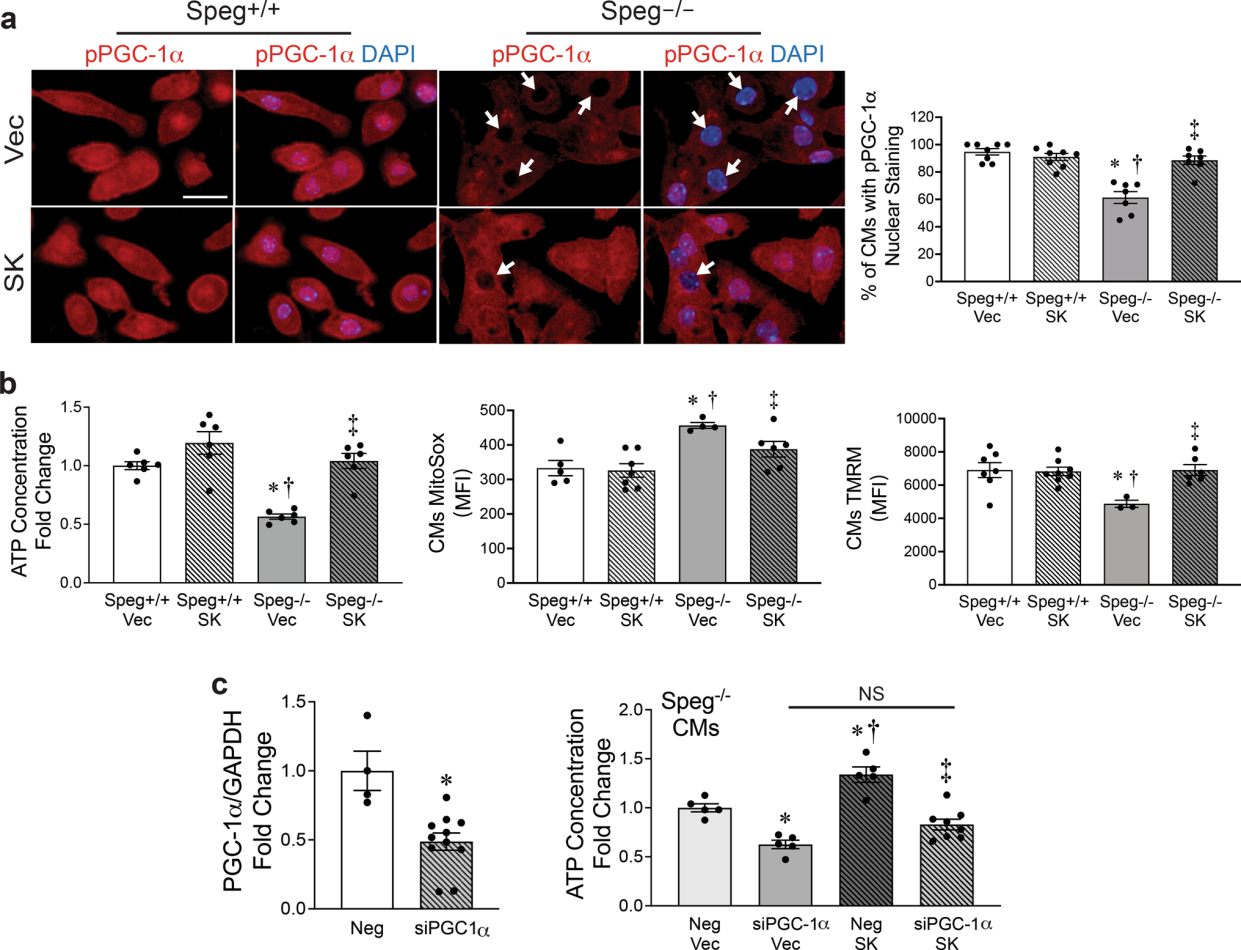
Speg SK rescues mitochondrial function in *Speg*^−/−^ CMs in part by phosphorylating PGC-1α in vitro. *Speg*^+/+^ and *Speg*^−/−^ CMs from E18.5 neonates were transfected with pFLAG-CMV-2 vector (Vec) or Speg SK. **a** Representative images of immunofluorescent staining for PGC-1α (red) and DAPI (blue, nuclei) in CMs. White arrows point to nuclei that do not stain for pPGC-1α. The bar graph depicts percentage of nuclei stained for pPGC-1α compared with total nuclei in bar graph (bottom), *n* = 7–8 for each group. **b** Quantitation of ATP concentration (left), mitoSox (middle) and TMRM (right) in CMs, shown as fold change to *Speg*^+/+^ CMs transfected with Vec, *n* = 3–8 for each group. **c** qRT-PCR for PGC-1α expression in CMs transfected by negative control (Neg) or siRNA-PGC-1α (siPGC-1α), *n* = 4–11 for each group (left). Quantitation of ATP concentration in *Speg*^−/−^ CMs that were co-transfected with Neg or siPGC-1α (right), and following transfection with either Vec or SK, *n* = 5–8 for each group. The data presented in bar graphs are displayed as mean ± SEM. One-way ANOVA was performed for multiple group comparisons (**a**, **b** and **c**), with *p* ≤ 0.0145, * versus *Speg*^+/+^ Vec or *Speg*^−/−^ Neg/Vec, † versus Speg^+/+^ SK or *Speg*^−/−^ siPGC-1α/Vec, and ‡ versus *Speg*^−/−^ Vec or *Speg*^−/−^ Neg/SK. *t*-test was performed for comparison of two groups in **c** (left), *p* = 0.0020, with * versus Neg. *NS* not significantly different. Scale bar represents 20μm in **a**

*Speg*^−/−^ and *Speg*^+/+^ E18.5 CMs were transfected with Vec or SK and stained for pPGC-1α. In the majority of *Speg*^+/+^ CMs transfected with either Vec or SK, staining for pPGC-1α showed stronger nuclear than cytoplasmic staining, with no significant difference between the two groups (Fig. 7a). Interestingly, in *Speg*^−/−^ CMs receiving Vec, significantly fewer cells demonstrated this stronger nuclear rather than cytoplasmic staining for pPGC-1α, compared with *Speg*^+/+^ CMs transfected with Vec or SK. However, SK transfection increased nuclear staining of pPGC-1α in *Speg*^−/−^ CMs, restoring it to levels analogous to the *Speg*^+/+^ CMs (Fig. 7a). ATP concentrations, MitoSox, and TMRM were next measured to assess mitochondrial function. *Speg*^+/+^ CMs transfected with SK showed a slightly higher ATP concentration compared with *Speg*^+*/*+^ CMs receiving Vec, but this change was not significantly different. ATP levels were significantly lower in *Speg*^−/−^ CMs transfected with Vec compared with *Speg*^+/+^ cells receiving Vec or SK. Notably, SK rescued ATP production in *Speg*^−/−^ CMs to levels comparable to those in the *Speg*^+/+^ CMs (Fig. 7b. left). Flow cytometry analysis revealed a decrease in MitoSox and an increase in TMRM in the *Speg*^−/−^ CMs transfected with SK compared with *Speg*^−/−^ CMs transfected with Vec (Fig. 7b, middle and right panels, respectively). Transfection of SK into the *Speg*^−/−^ CMs increased the membrane potential and decreased superoxide production to levels analogous to *Speg*^+/+^ CMs.

To understand whether the effects of Speg on mitochondrial function were specifically dependent on PGC-1α, siPGC-1α was utilized to silence PGC-1α in CMs. CMs transfected with siPGC-1α demonstrated an ~ 51% reduction in mRNA levels of PGC-1α (Fig. 7c, left). Silencing PGC-1α in *Speg*^−/−^ CMs led to a further reduction in ATP concentration compared to *Speg*^−/−^ CMs transfected with a non-targeting siRNA (Neg). Overexpression of SK in *Speg*^−/−^ CMs significantly increased ATP levels compared with *Speg*^−/−^ CMs transfected with Neg + Vec and siPGC-1α + Vec. However, Speg SK increased ATP concentrations in *Speg*^−/−^ CMs was abolished when PGC-1α was silenced (Fig. 7c, right). Taken together, these data reveal that Speg SK rescues mitochondrial ATP production in a PGC-1α-dependent manner.

## Discussion

Speg, a member of the MLCK family of proteins, has been reported to play a role in the myofibrillar structure and myocardial contractility in fetal and adult hearts, through phosphorylating sarcomeric proteins or proteins that mediate calcium homeostasis [6, 7, 24, 30]. The present study is the first to demonstrate that Speg also plays an important role in the structure and function of mitochondria during fetal development. Disruption of Speg results in abnormal cristae structure of the inner mitochondrial membrane, which corresponds with decreased ATP production, increased oxidative stress, and depolarization of the mitochondrial membrane potential. The heart has the highest ATP consumption per tissue mass in the body, but relatively limited energy reserves [40]. In other words, the amount of ATP generated and used per minute is many times greater than the size of the ATP storage pool. Therefore, maintaining an adequate ATP supply is critical for cardiac performance [19]. Interestingly, impaired mitochondria in embryonic hearts results in cardiomyopathy and fetal/neonatal lethality in animal models and human patients [48]. A 25–30% reduction in ATP production has been reported in heart failure, suggestive of advanced disease [4, 19, 35, 45]. The energy demands of the heart increase dramatically at birth, and so mitochondrial density also increases sharply during the perinatal period [5, 31]. Our study demonstrated that mitochondria ATP production from either glucose or palmitate (long-chain fatty acid) was decreased, contributing to a 27 ± 8.7% reduction in ATP concentration in E18.5 *Speg*^−/−^ CMs, which was associated with mitochondrial cristae abnormalities. All together, we propose that reduced myocardial contractility, due to insufficient ATP production and abnormal myofibril structure in immature CMs [29, 30, 44], contributes to neonatal mortality in the presence of Speg deficiency [29, 30].

The vast majority of ATP synthesis occurs via oxidative phosphorylation in the mitochondrial cristae, which are folds of the inner mitochondrial membrane containing the electron transport system. The cristae provide a large amount of surface area for the chemical reactions of the electron transport chain to produce ATP [17, 48]. Our data demonstrate that mitochondrial cristae in *Speg*^*−/−*^ CMs are not only morphologically abnormal, but also reduced in number and percentage area compared to *Speg*^+*/*+^ CMs. Very immature mitochondria (class I) with no or few cristae are observed in E8.5 CMs and evolve to immature mitochondria (class II) around E10.5. Class II mitochondria exhibit few tubular cristae connecting the peripheral inner boundary membrane. Neither class I nor II mitochondria create the coupling between the electron transport chain activity and ATP generation [48]. At E13.5 through the perinatal period, near mature mitochondria (class III) with increased tubular cristae and mature mitochondria (class IV) with abundant tubular cristae, highly compacted matrices, and multiple tubular connections to the periphery occupy CMs [17, 48]. The most striking alteration in mitochondrial morphology in *Speg*^−/−^ CMs are the abnormalities in cristae. Almost 90% of mitochondria are class I and II in *Speg*^−/−^ hearts, while *Speg*^+/+^ hearts are occupied by 90% mature class III and IV mitochondria at E18.5. These data suggest there is a defect in mitochondrial maturation in *Speg*^−/−^ hearts. Previously we showed that *Speg*^−/−^ cardiac progenitor cells fail to differentiate into mature CMs [29, 30], a phenotype which is very similar to induced pluripotent stem cell-derived CMs, with an immature or fetal-like phenotype [13, 17]. Mitochondrial biogenesis, dynamics, and mitophagy play key roles in the maturation of induced pluripotent stem cell-derived CMs [13, 17]. In our study, decreased mitochondrial biogenesis and function occurred in *Speg*^−/−^ hearts, which began to enlarge as early at E16.5 with evidence of a dilated cardiomyopathy by E18.5 [29, 30]. Taken together, these data suggest that mitochondrial abnormalities in structure and function contribute to CM immaturity in *Speg*^−/−^ hearts leading to dysfunction [12, 15].

The heart is the first organ to develop at E7.5 in mice [41, 48]. Mitochondria are one of the earliest organelles in CMs of mouse embryonic hearts, which can be observed at E8.5–9.5 [48]. The present study shows that Speg was expressed in the first and second heart fields, as early as E7.5 at the beginning of heart development, and is restricted to CMs, with no differences in spatial distribution during development [29, 30]. At the same time, PGC-1α, a key regulator for mitochondrial development, also appears in embryonic hearts and precedes the expression of mitochondrial markers. Recent evidence revealed that Speg phosphorylates SERCA2a and RYR2 of SR to modulate calcium handling and maintain calcium homeostasis in adult hearts [6, 7, 24, 26, 36]. However, during development, when less nuclear expression of pPGC-1α was evident in *Speg*^*−/−*^ hearts at E9.5, SR and t-tubules were not yet detectable. Moreover, SERCA2a and RyR2 are expressed at low levels during the early embryonic period[23, 39], and t-tubules are absent in early fetal CMs and are present only as small indentations of the sarcolemma in late fetal CMs, as t-tubules are not fully developed until ~ 2–3 weeks after birth [27, 42, 49]. Finally, the mitochondrial calcium concentrations showed no differences, and there were no alterations in mPTP opening in *Speg*^*−/−*^ compared with *Speg*^+*/*+^ CMs at E18.5. Taken together, these data suggest mitochondrial abnormalities induced by Speg disruption appear to be a fundamental factor in the developmental defects of fetal hearts, and this goes beyond impaired calcium homeostasis.

PGC-1α is a well-known central factor in mitochondrial development and function. Posttranslational phosphorylation promotes the activity of PGC-1α, followed by translocation of PGC-1α to the nucleus to bind NUGEMPs [9, 40, 47]. Our data demonstrated that phosphorylation of PGC-1α and the ratio of phosphorylated to total PGC-1α were reduced in *Speg*^*−/−*^ hearts, resulting in downregulation of its downstream genes including NRF1, ERRα, β andγ, and PPRGα. PGC-1α is well known to be phosphorylated by P38 MAPK, calcineurin, AMPK, c-AMP, cGMP, and CREB[9, 40, 47]. Interestingly, none of these PGC-1α upstream proteins were found to be altered in *Speg*^*−/−*^ hearts, suggesting that Speg may have a direct effect on PGC-1α. In addition, co-immunoprecipitation analysis revealed that Speg forms a complex with PGC-1α. Immunofluorescent co-staining further showed that Speg not only overlapped with expression of sarcomeric α-actinin at the z-lines, but also partially co-localized with pPGC-1α between the z-lines indicating that Speg anchors at the z-line and extends reticular branches to bind and phosphorylate PGC-1α. We also found that pPGC-1α has less nuclear staining in *Speg*^*−/−*^ CMs, whereas overexpression of Speg SK increased pPGC-1α in the nucleus, resulting in restoration of ATP production and mitochondrial membrane potential, and decreased production of reactive oxygen species in *Speg*^*−/−*^ CMs. Silencing of PGC-1α in *Speg*^*−/−*^ CMs abolished the ATP production rescued by Speg SK overexpression. These data suggest that Speg interacts with and phosphorylates PGC-1α in the complex, and that translocation of pPGC-1α into the nucleus results in increased ATP production.

We believe that mitochondrial abnormalities induced by disruption of Speg is a fundamental factor in the developmental defects of fetal hearts and contributed to immature CMs. However additional experiments need to be performed to exclude impaired calcium homeostasis. Furthermore, Speg has two tandemly arranged serine/threonine kinase domains. In this study, we explored the Speg SK domain using an expression plasmid that encompasses amino acid residues 1598–1862 of the protein. Binding domains within this region of the Speg protein include the active site (position 1724) and ATP binding sites (positions 1612–1620, and 1635). The active site is the region where a substrate binds and catalysis of the kinase reaction occurs, and our SK expression plasmid was functionally active by phosphorylating PGC-1α, which contributed to the improved mitochondrial function in vitro. It is feasible that the other SK domain of the tandem may also contribute to the impact of Speg on mitochondrial function, and this will need to be explored in future studies.

In conclusion, Speg is expressed in the heart prior to the expression of other mitochondrial specific genes, as early as E7.5. Disruption of Speg results in immature mitochondria with no or few cristae and abnormal morphology, corresponding with reduced mitochondrial function, including less ATP production. Speg complexes with PGC-1α to promote phosphorylation, thereby rescuing ATP production, in a PGC-1α -dependent manner. These data demonstrate for the first time that Speg plays a critical role in the structure and function of mitochondria during cardiac development.

### Supplementary Information

Below is the link to the electronic supplementary material.

Supplementary file1 (DOCX 19620 KB)

## Data Availability

The authors declare that the data supporting the findings of this study are available within the article, and in the supplementary information files.

## Abbreviations

ACTN2: Actinin alpha 2
AMPK: Adenosine monophosphate-activated protein kinase
CaMK: Calcium/calmodulin-dependent protein kinase
CAV3: Caveolin-3
CCCP: Carbonyl cyanide m-chlorophenyl hydrazone
CMs: Cardiomyocytes
CREB: C-AMP-response element binding protein
DAPI: 4′,6-Diamidino-2-phenylindole
ECAR: Extracellular acidification rate
ERR: Estrogen-related receptor
FAO: Fatty acid oxidation
GSK3β: Glycogen synthase kinase 3β
JC-1: Tetraethylbenzimidazolylcarbocyanine iodide
MAPK: P38 mitogen-activated protein kinase
MFI: Mean fluorescence intensity
MLCK: Myosin light chain kinase
MOSTA: Spatial Transcriptomic Data Set
mPTP: Mitochondrial permeability transition pore
MCU: The mitochondrial calcium uniporter
NAO: Nonyl acridine orange
NCLX: Mitochondrial Na^+^/Ca2^+^/Li^+^ exchanger
NRF1/2: Nuclear respiratory factor 1/2
NUGEMPs: Nuclear genes encoding mitochondrial proteins
OCR: Oxygen consumption rate
Oligo: Oligomycin
PGC-1: Peroxisome proliferator-activated receptor γ coactivator 1
PPAR: Peroxisome proliferator-activated receptor
qRT-PCR: Quantitative real-time PCR
ROS: Reactive oxygen species
RyR2: Ryanodine receptor 2
SERCA2a: Sarcoplasmic reticulum Ca2^+^ ATPase 2a
SK: Serine/threonine kinase domain
SPEG: Striated preferentially expressed gene
TEM: Transmission electron microscopy
TMRM: Tetramethylrhodamine methyl ester
TNNT2: Cardiac troponin T2
TOM20: Mitochondrial membrane complex subunit 20
TUNEL: Terminal deoxynucleotidyl transferase dUTP nick end labeling
WGA: Wheat germ agglutinin
VDAC: Voltage-dependent anion-selective channel

## References

[1] Agrawal PB, Pierson CR, Joshi M, Liu X, Ravenscroft G, Moghadaszadeh B, Talabere T, Viola M, Swanson LC, Haliloglu G, Talim B, Yau KS, Allcock RJ, Laing NG, Perrella MA, Beggs AH (2014). SPEG interacts with myotubularin, and its deficiency causes centronuclear myopathy with dilated cardiomyopathy. Am J Hum Genet.

[2] Angelini A, Pi X, Xie L (2022). Evaluation of long-chain fatty acid respiration in neonatal mouse cardiomyocytes using seahorse instrument. STAR Protoc.

[3] Bartelds B, Knoester H, Smid GB, Takens J, Visser GH, Penninga L, van der Leij FR, Beaufort-Krol GC, Zijlstra WG, Heymans HS, Kuipers JR (2000). Perinatal changes in myocardial metabolism in lambs. Circulation.

[4] Bottomley PA, Panjrath GS, Lai S, Hirsch GA, Wu K, Najjar SS, Steinberg A, Gerstenblith G, Weiss RG (2013). Metabolic rates of ATP transfer through creatine kinase (CK Flux) predict clinical heart failure events and death. Sci Transl Med.

[5] Buroker NE, Ning XH, Portman M (2008). Cardiac PPARalpha protein expression is constant as alternate nuclear receptors and PGC-1 coordinately increase during the postnatal metabolic transition. PPAR Res.

[6] Campbell H, Aguilar-Sanchez Y, Quick AP, Dobrev D, Wehrens XHT (2021). SPEG: a key regulator of cardiac calcium homeostasis. Cardiovasc Res.

[7] Campbell HM, Quick AP, Abu-Taha I, Chiang DY, Kramm CF, Word TA, Brandenburg S, Hulsurkar M, Alsina KM, Liu HB, Martin B, Uhlenkamp D, Moore OM, Lahiri SK, Corradini E, Kamler M, Heck AJR, Lehnart SE, Dobrev D, Wehrens XHT (2020). Loss of SPEG inhibitory phosphorylation of ryanodine receptor type-2 promotes atrial fibrillation. Circulation.

[8] Chen A, Liao S, Cheng M, Ma K, Wu L, Lai Y, Qiu X, Yang J, Xu J, Hao S, Wang X, Lu H, Chen X, Liu X, Huang X, Li Z, Hong Y, Jiang Y, Peng J, Liu S, Shen M, Liu C, Li Q, Yuan Y, Wei X, Zheng H, Feng W, Wang Z, Liu Y, Wang Z, Yang Y, Xiang H, Han L, Qin B, Guo P, Lai G, Munoz-Canoves P, Maxwell PH, Thiery JP, Wu QF, Zhao F, Chen B, Li M, Dai X, Wang S, Kuang H, Hui J, Wang L, Fei JF, Wang O, Wei X, Lu H, Wang B, Liu S, Gu Y, Ni M, Zhang W, Mu F, Yin Y, Yang H, Lisby M, Cornall RJ, Mulder J, Uhlen M, Esteban MA, Li Y, Liu L, Xu X, Wang J (2022). Spatiotemporal transcriptomic atlas of mouse organogenesis using DNA nanoball-patterned arrays. Cell.

[9] Dorn GW, Vega RB, Kelly DP (2015). Mitochondrial biogenesis and dynamics in the developing and diseased heart. Genes Dev.

[10] Ebihara T, Nagatomo T, Sugiyama Y, Tsuruoka T, Osone Y, Shimura M, Tajika M, Matsuhashi T, Ichimoto K, Matsunaga A, Akiyama N, Ogawa-Tominaga M, Yatsuka Y, Nitta KR, Kishita Y, Fushimi T, Imai-Okazaki A, Ohtake A, Okazaki Y, Murayama K (2021). Neonatal-onset mitochondrial disease: clinical features, molecular diagnosis and prognosis. Arch Dis Child Fetal Neonatal Ed.

[11] Eom S, Lee HN, Lee S, Kang HC, Lee JS, Kim HD, Lee YM (2017). Cause of death in children with mitochondrial diseases. Pediatr Neurol.

[12] Folmes CD, Dzeja PP, Nelson TJ, Terzic A (2012). Mitochondria in control of cell fate. Circ Res.

[13] Garbern JC, Lee RT (2021). Mitochondria and metabolic transitions in cardiomyocytes: lessons from development for stem cell-derived cardiomyocytes. Stem Cell Res Ther.

[14] Greutmann M, Tobler D (2012). Changing epidemiology and mortality in adult congenital heart disease: looking into the future. Future Cardiol.

[15] Guo Y, Pu WT (2020). Cardiomyocyte maturation: new phase in development. Circ Res.

[16] Gustafsson AB, Gottlieb RA (2008). Heart mitochondria: gates of life and death. Cardiovasc Res.

[17] Hom JR, Quintanilla RA, Hoffman DL, de Mesy Bentley KL, Molkentin JD, Sheu SS, Porter GA (2011). The permeability transition pore controls cardiac mitochondrial maturation and myocyte differentiation. Dev Cell.

[18] Hsieh CM, Fukumoto S, Layne MD, Maemura K, Charles H, Patel A, Perrella MA, Lee ME (2000). Striated muscle preferentially expressed genes alpha and beta are two serine/threonine protein kinases derived from the same gene as the aortic preferentially expressed gene-1. J Biol Chem.

[19] Ingwall JS, Weiss RG (2004). Is the failing heart energy starved? On using chemical energy to support cardiac function. Circ Res.

[20] Kamisago M, Sharma SD, DePalma SR, Solomon S, Sharma P, McDonough B, Smoot L, Mullen MP, Woolf PK, Wigle ED, Seidman JG, Seidman CE (2000). Mutations in sarcomere protein genes as a cause of dilated cardiomyopathy. N Engl J Med.

[21] Kampourakis T, Sun YB, Irving M (2016). Myosin light chain phosphorylation enhances contraction of heart muscle via structural changes in both thick and thin filaments. Proc Natl Acad Sci U S A.

[22] Karbassi E, Fenix A, Marchiano S, Muraoka N, Nakamura K, Yang X, Murry CE (2020). Cardiomyocyte maturation: advances in knowledge and implications for regenerative medicine. Nat Rev Cardiol.

[23] Kawamura Y, Ishiwata T, Takizawa M, Ishida H, Asano Y, Nonoyama S (2010). Fetal and neonatal development of Ca2+ transients and functional sarcoplasmic reticulum in beating mouse hearts. Circ J.

[24] Kusumoto D, Yuasa S, Fukuda K (2019). SPEG, an indispensable kinase of SERCA2a for calcium homeostasis. Circ Res.

[25] Lehman JJ, Barger PM, Kovacs A, Saffitz JE, Medeiros DM, Kelly DP (2000). Peroxisome proliferator-activated receptor gamma coactivator-1 promotes cardiac mitochondrial biogenesis. J Clin Invest.

[26] Levitas A, Muhammad E, Zhang Y, Perea Gil I, Serrano R, Diaz N, Arafat M, Gavidia AA, Kapiloff MS, Mercola M, Etzion Y, Parvari R, Karakikes I (2020). A novel recessive mutation in SPEG causes early onset dilated cardiomyopathy. PLoS Genet.

[27] Liau B, Zhang D, Bursac N (2012). Functional cardiac tissue engineering. Regen Med.

[28] Liesa M, Shirihai OS (2013). Mitochondrial dynamics in the regulation of nutrient utilization and energy expenditure. Cell Metab.

[29] Liu X, Hall SRR, Wang Z, Huang H, Ghanta S, Di Sante M, Leri A, Anversa P, Perrella MA (2015). Rescue of neonatal cardiac dysfunction in mice by administration of cardiac progenitor cells in utero. Nat Commun.

[30] Liu X, Ramjiganesh T, Chen YH, Chung SW, Hall SR, Schissel SL, Padera RF, Liao R, Ackerman KG, Kajstura J, Leri A, Anversa P, Yet SF, Layne MD, Perrella MA (2009). Disruption of striated preferentially expressed gene locus leads to dilated cardiomyopathy in mice. Circulation.

[31] Lopaschuk GD, Collins-Nakai RL, Itoi T (1992). Developmental changes in energy substrate use by the heart. Cardiovasc Res.

[32] Luo S, Rosen SM, Li Q, Agrawal PB (2021). Striated preferentially expressed protein kinase (SPEG) in muscle development, function, and disease. Int J Mol Sci.

[33] Luo X, Liao C, Quan J, Cheng C, Zhao X, Bode AM, Cao Y (2019). Posttranslational regulation of PGC-1alpha and its implication in cancer metabolism. Int J Cancer.

[34] McNally EM, Golbus JR, Puckelwartz MJ (2013). Genetic mutations and mechanisms in dilated cardiomyopathy. J Clin Invest.

[35] Nascimben L, Ingwall JS, Pauletto P, Friedrich J, Gwathmey JK, Saks V, Pessina AC, Allen PD (1996). Creatine kinase system in failing and nonfailing human myocardium. Circulation.

[36] Quan C, Du Q, Li M, Wang R, Ouyang Q, Su S, Zhu S, Chen Q, Sheng Y, Chen L, Wang H, Campbell DG, MacKintosh C, Yang Z, Ouyang K, Wang HY, Chen S (2020). A PKB-SPEG signaling nexus links insulin resistance with diabetic cardiomyopathy by regulating calcium homeostasis. Nat Commun.

[37] Quick AP, Wang Q, Philippen LE, Barreto-Torres G, Chiang DY, Beavers D, Wang G, Khalid M, Reynolds JO, Campbell HM, Showell J, McCauley MD, Scholten A, Wehrens XH (2017). SPEG (striated muscle preferentially expressed protein kinase) is essential for cardiac function by regulating junctional membrane complex activity. Circ Res.

[38] Quiros PM, Goyal A, Jha P, Auwerx J (2017). Analysis of mtDNA/nDNA ratio in mice. Curr Protoc Mouse Biol.

[39] Ribadeau-Dumas A, Brady M, Boateng SY, Schwartz K, Boheler KR (1999). Sarco(endo)plasmic reticulum Ca(2+)-ATPase (SERCA2) gene products are regulated post-transcriptionally during rat cardiac development. Cardiovasc Res.

[40] Rowe GC, Jiang A, Arany Z (2010). PGC-1 coactivators in cardiac development and disease. Circ Res.

[41] Schleich JM, Abdulla T, Summers R, Houyel L (2013). An overview of cardiac morphogenesis. Arch Cardiovasc Dis.

[42] Scuderi GJ, Butcher J (2017). Naturally engineered maturation of cardiomyocytes. Front Cell Dev Biol.

[43] Segawa M, Wolf DM, Hultgren NW, Williams DS, van der Bliek AM, Shackelford DB, Liesa M, Shirihai OS (2020). Quantification of cristae architecture reveals time-dependent characteristics of individual mitochondria. Life Sci Alliance.

[44] Shu C, Huang H, Xu Y, Rota M, Sorrentino A, Peng Y, Padera RF, Huntoon V, Agrawal PB, Liu X, Perrella MA (2018). Pressure overload in mice with haploinsufficiency of striated preferentially expressed gene leads to decompensated heart failure. Front Physiol.

[45] Starling RC, Hammer DF, Altschuld RA (1998). Human myocardial ATP content and in vivo contractile function. Mol Cell Biochem.

[46] Tobita T, Nomura S, Morita H, Ko T, Fujita T, Toko H, Uto K, Hagiwara N, Aburatani H, Komuro I (2017). Identification of MYLK3 mutations in familial dilated cardiomyopathy. Sci Rep.

[47] Uguccioni G, D'Souza D, Hood DA (2010). Regulation of PPARgamma coactivator-1alpha function and expression in muscle: effect of exercise. PPAR Res.

[48] Zhao Q, Sun Q, Zhou L, Liu K, Jiao K (2019). Complex regulation of mitochondrial function during cardiac development. J Am Heart Assoc.

[49] Ziman AP, Gomez-Viquez NL, Bloch RJ, Lederer WJ (2010). Excitation-contraction coupling changes during postnatal cardiac development. J Mol Cell Cardiol.

