# Supplementary material for: Striated preferentially expressed gene deficiency leads to mitochondrial dysfunction in developing cardiomyocytes
Source: Basic Res Cardiol. 2023 Dec 26;119(1):151–68. doi: 10.1007/s00395-023-01029-7 (PMC10837246; doi:10.1007/s00395-023-01029-7)
Supplement: Supplementary file 1 — Supplementary file1 (DOCX 19620 KB) [file 395_2023_1029_MOESM1_ESM.docx]

**Supplementary Information**

| **Table 1. Mouse Primer** |  |  |
| --- | --- | --- |
| Mouse Primer for qRT-PCR | Forward | Reverse |
| Speg | 5’-AATGTCCTGGGCAGCATTAC-3’ | 5’-TGCTGTGTCATGGTAGGTCTG-3’ |
| mtDNA | 5’-CCTATCACCCTTGCCATCAT-3’ | 5’-GAGGCTGTTGCTTGTGTGAC-3’ |
| nDNA | 5’-ATGGAAAGCCTGCCATCATG-3’ | 5’-TCCTTGTTGTTCAGCATCAC-3’ |
| PGC-1α | 5’-GGACATGTGCAGCCAAGACTCT-3’ | 5’-CACTTCAATCCACCCAGAAAGCT-3’ |
| PGC-1β | 5’-AGTCAGCGGCCTTGTGTCAA-3’ | 5’-ACTCTGGGACAGGGCAGCA-3’ |
| NRF-1 | 5’-GCACCTTTGGAGAATGTGGT-3’ | 5’-GGGTCATTTTGTCCACAGAGA-3’ |
| NRF-2 | 5’-CCAGCTACTCCCAGGTTGC-3’ | 5’-CCTGATGAGGGGCAGTGA-3’ |
| PPAR-α | 5’-TCGGCGAACTATTCGGCTG-3’ | 5’-GCACTTGTGAAAACGGCAGT-3’ |
| PPAR-β/δ | 5’-TTGAGCCCAAGTTCGAGTTTG-3’ | 5’-CGGTCTCCACACAGAATGATG-3’ |
| PPAR-γ | 5’-TGTGGGGATAAAGCATCAGGC-3’ | 5’-CCGGCAGTTAAGATCACACCTAT-3’ |
| ERR-α | 5’-CCAATGAGTGTGAGATCACCG-3’ | 5’-CCGTTTGTACTTCTGCCGTCG-3’ |
| ERR-β | 5’-CAGATCGGGAGCTTGTGTTC-3’ | 5’-TGGTCCCCAAGTGTCAGACT-3’ |
| ERR-γ | 5’-CTGACGGACAGCGTCAACC-3’ | 5’-GGCGAGTCAAGTCCGTTCTG-3’ |
| GAPDH | 5’-CCTGGAGAAACCTGCCAAG-3’ | 5’-AGGAGACAACCTGGTCCTCA-3’ |
| Mouse Primer for genotyping (PCR) | Forward | Reverse |
| Speg primers | 5’-GCTGAGGAATCGACAGCCCG-3’ | 5’-TGCCGAGCGCCATATTCGTT-3’ |
| Neomycin primers | 5’-CGTTGGCTACCCGTGATATT-3’ | 5’-AGGCGATAGAAGGCGATGCG-3’ |

| **Table 2. Primary antibody** | |  |  |  |
| --- | --- | --- | --- | --- |
| Name | Company | Category ID | Concentration | Used for |
| β-Galactosidase | Abcam | ab9361 | 1:200 | Immunohistochemistry staining |
| NCLX | Invitrogen | PA5-114330 | 1:100 | Immunofluorescent staining |
|  |  |  | 1:800 | Western Blot |
| S571 phospho-PGC-1α | R & D System | AF6650 | 1:200 | Western Blot |
|  |  |  | 1:200 | Immunofluorescent staining |
| PGC-1α | Millipore Sigma | AB3242 | 1:1000 | Western Blot |
|  |  |  | 1:50 | Immunoprecipitation |
|  | Abcam | ab54481 | 1:100 | Immunofluorescent staining |
|  | Abcam | ab106814 | 1:100 | Immunofluorescent staining |
| APEG-1/SPEG | Mark Perrella's Lab | Ref#18 | 1:500 | Western Blot |
|  |  |  | 1:20 | Immunoprecipitation |
|  |  |  | 1:100 | Immunofluorescent staining |
| Flag M2 | Cell Signaling | 2368S | 1:200 | Immunofluorescent and flow cytometry staining |
| Rabbit anti-mouse IgG | Cell Signaling | 2729S | 1:150 | Immunoprecipitation |
| Sar α-Actinin | Millipore Sigma | A7811 | 1:200 | Immunofluorescent staining |
| FLAG M2 | Cell Signaling | 2368 | 1:1000 | Western Blot |
| DAPI | Millipore Sigma | D9542 | 1:1000 | Immunofluorescent staining |
| GAPDH | Cell Signaling | 3683 | 1:1000 | Western Blot |
| TOM20 | Santa Cruz | sc-11415 | 1:50 | Immunofluorescent staining |
| CAV3 | R & D System | MAB6706 | 1:50 | Immunofluorescent staining |
| SERCA2α | Cell Signaling | 9580S | 1:50 | Immunofluorescent staining |
| MCU | Cell Signaling | 14997S | 1:1000 | Western Blot |
| VDAC1 | Abcam | ab14734 | 1:1000 | Western Blot |
| Cleaved/total caspase 3 | Cell Signaling | 9665S | 1:1000 | Western Blot |
| Cleaved/total PARP | Cell Signaling | 9544P | 1:1000 | Western Blot |
| ApoAlert™ Tunnel Staining | Clontech | 630107 | 1/50 （v/v） | TUNEL assay |
| T286 phospho-CaMKII | Cell Signaling | 3361 | 1:500 | Western Blot |
| CaMKII | Cell Signaling | 4436 | 1:1000 | Western Blot |
| T180/T182 phospho-p38 MAPK | Cell Signaling | 4511 | 1:500 | Western Blot |
| p38 MAPK | Cell Signaling | 9212 | 1:1000 | Western Blot |
| S473 phospho-AKT | Cell Signaling | 4060S | 1:500 | Western Blot |
| AKT | Cell Signaling | 4691 | 1:1000 | Western Blot |
| S9 phospho-GSK-3b | Cell Signaling | 9336 | 1:500 | Western Blot |
| GSK-3b | Cell Signaling | 9315 | 1:1000 | Western Blot |
| T197 phospho-PKA C | Cell Signaling | 5661 | 1:500 | Western Blot |
| PKA | Cell Signaling | 5842 | 1:1000 | Western Blot |
| SIRT1 | Abcam | ab110304 | 1:1000 | Western Blot |
| AMPKa1/2 | Santa Cruz | sc-74461 | 1:1000 | Western Blot |
| Calcineurin (CaN) | Cell Signaling | 2614 | 1:1000 | Western Blot |

| **Table 3. Reagents** |  |  |
| --- | --- | --- |
| Name | Company | Category ID |
| Neonatal cadiomyocyte culture medium | Cellutron | M-8031 |
| Neonatal cardiomyocyte isolation kit | Cellutron | nc-6031 |
| PierceTM IP lysis buffer | Thermo Scientific | 87787 |
| Protease inhibitor | Millipore Sigma | 11836153001 |
| Protein-A Dynabeads | Invitrogen | 10001D |
| Phosphatase inhibitor | Millipore Sigma | P0044, P5726 |
| Luminescent ATPlite assay system | Perkin Elmer | 6016943 |
| Seahorse XF Cell Mito Stress Test Kit | Agilent Tech Inc | 103015-100 |
| Seahorse XF Palmitate-BSA FAO Substrate kit | Agilent Tech Inc | 102720-100 |
| Seahorse XF DMEM assay medium pack | Agilent Tech Inc | 103680-100 |
| Seahorse XF Calibrant Solution | Agilent Tech Inc | 100840-000 |
| L-Carnitine hydrochloride synt | Fisher Scientific | 501790101 |
| DMEM without glucose | Fisher Scientific | 14430-01 |
| Ppargc1α Mouse siRNA Oligo Duplex (Locus ID 19017) | OriGene | SR427524 |
| Trilencer-27 Universal scrambled negative control siRNA duplex | OriGene | SR30004 |
| Opti-MEM Medium | Gibco | 51985 |
| Lipofectamine RNAiMAX Reagent | Invitrogen | 13778 |
| pFLAG-CMV-2-Speg internal serine/threonine kinase construct | Perrella Lab | Ref#34 |
| pCMV-FLAG-2 | Millipore Sigma | C6114 |
| Fugene HD | Promega | E5911 |
| Tetraethylbenzimidazolylcarbocyanine iodide (JC-1) | Invitrogen | M34152 |
| Tetramethylrhodamine, methyl ester (TMRM) | Thermo Scientific | M20036 |
| Tetramethylrhodamine, methyl ester (TMRM) | Invitrogen | I34361 |
| MitoSoxTM Red | Thermo Scientific | M36008 |
| Nonyl Acridine Orange (Acridine Orange 10-Nonyl Bromide) | Thermo Scientific | A1372 |
| Mito Tracker | Thermo Scientific | C1430 |
| Fluo-4 AM image kit | Thermofisher Sci | F10489 |
| Calcein AM | Thermofisher Sci | C3100MP |
| Cobalt Chloride Hexahydrate | Millipore Sigma | 7791-13-1 |


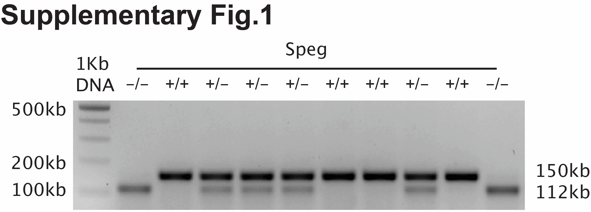


**Supplementary Figure 1. Speg genotyping by PCR.** The offsprings from the *Speg* heterozygous (+/-) breeding. The upper and lower bands present wild type (150kb) and Neomycin (112kb), respectively.


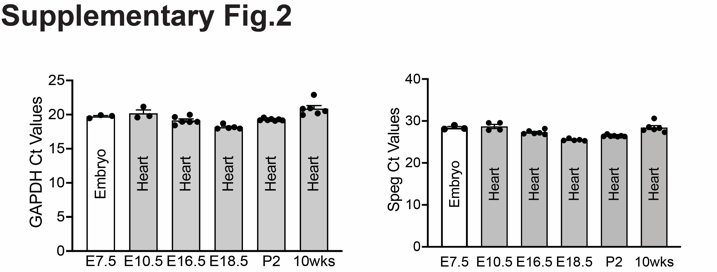


**Supplementary Figure 2. Speg and GAPDH expression.** Embryos (E) 7.5 and hearts (from E10.5 to 10-week-old) were harvested and qRT-PCR was performed. Quantitation of Ct values of GAPDH (upper panel) and Speg (lower panel) expression.

**
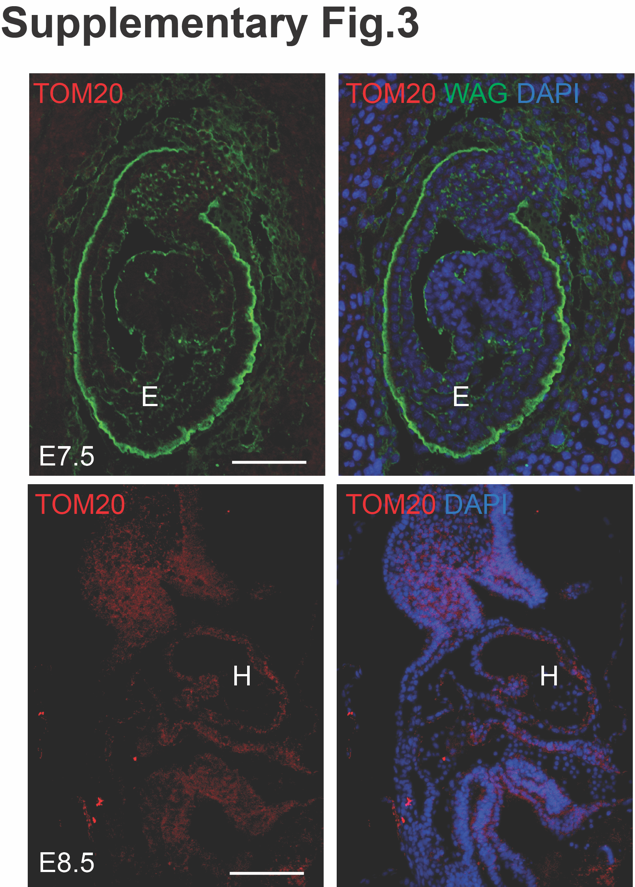
**

**Supplementary Figure 3. TOM20 expression in early developing mouse hearts.** Representative images of immunofluorescent staining for TOM20 (red), wheat germ agglutinin (WGA, green) conjugated with Alexa Fluor^TM^ 488, and DAPI (blue, nuclei) of embryos (E). H-heart. Scale bars represents 100μm.


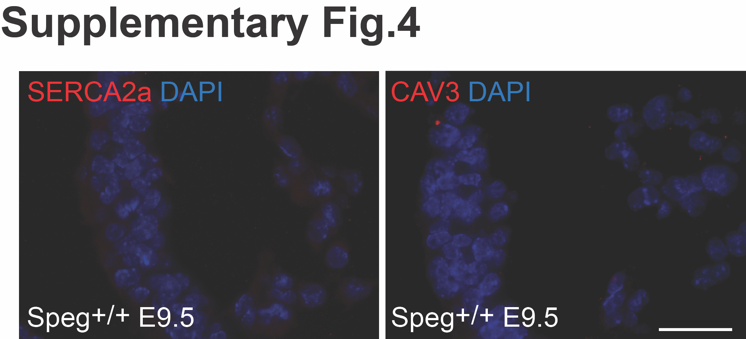


**Supplementary Figure 4. Sarcoplasmic reticulum and tubule were undetectable in E9.5 heart.** Representative images of immunofluorescent staining for SERCA2a and caveolin-3 (Cav3) (red) and DAPI for nuclear of embryos (E) heart. Scale bars represents 20μm.


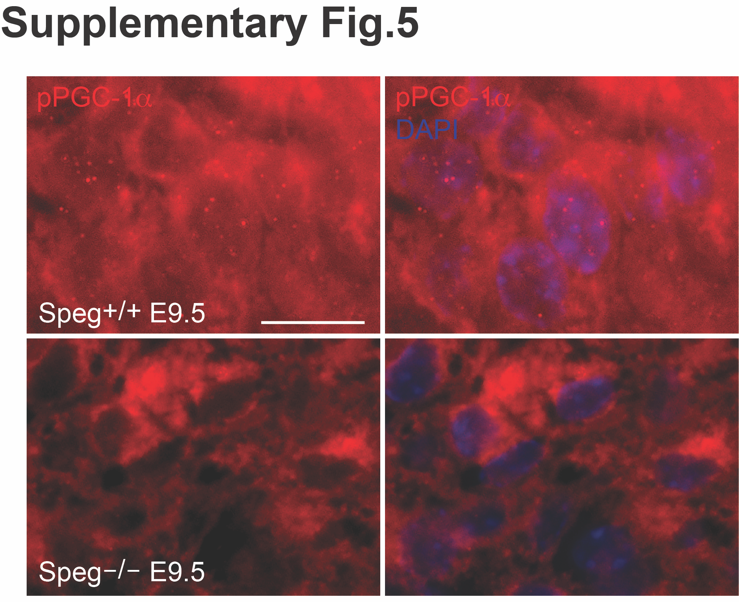


**Supplementary Figure 5. Decreased expression of pPGC-1α in nuclear of Speg^-/-^ heart.** Representative images of immunofluorescent staining for pPGC-1α (red) and DAPI for nuclear of embryos (E) hearts. Scale bars represents 10μm.


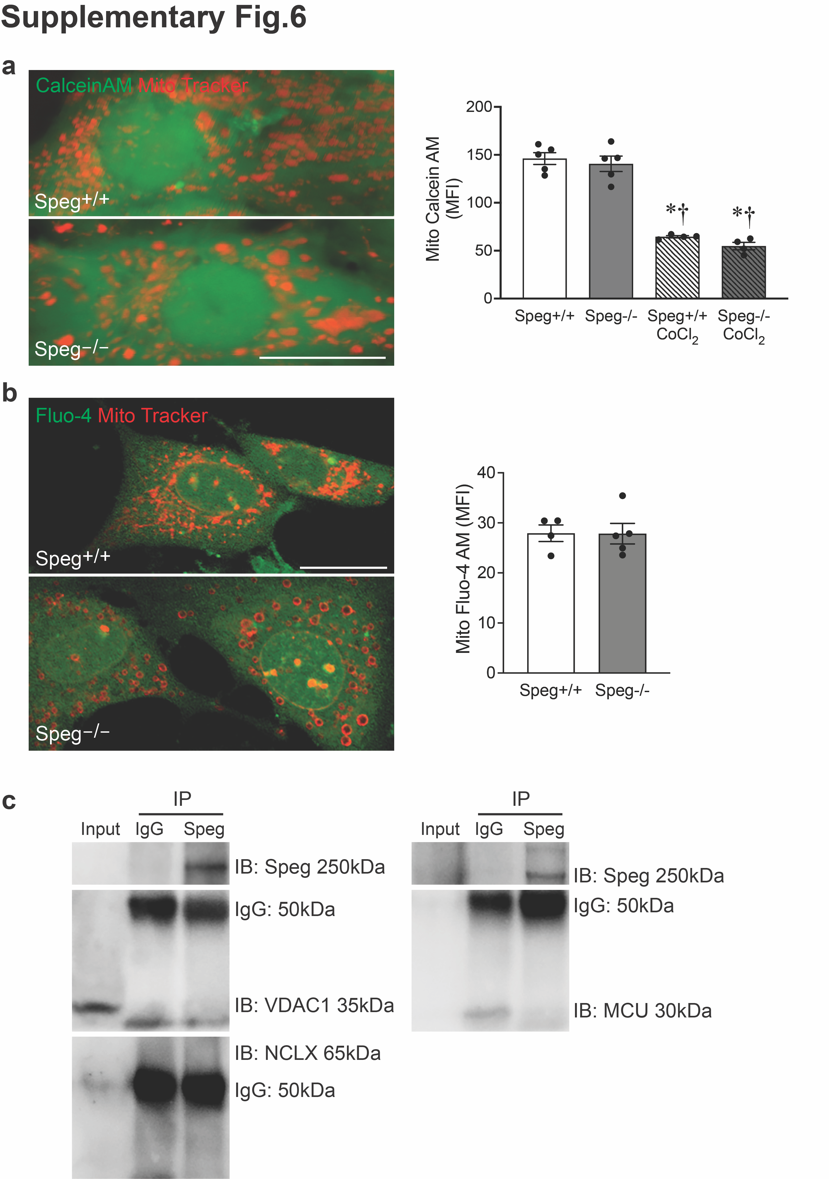


**Supplementary Figure 6. No alteration of mitochondria calcium in Speg^-/-^ CMs.**

E18.5 CMs were cultured *in vitro* and labeled by fluorescent dyes, following the manufacturer’s instruction. **a**) Representative images of co-staining for Calcein AM (green) and MitoTracker (red). The bar graph (right) shows quantitation (MFI), presented as mean±SEM. Cobalt chloride hexahydrate (CoCL_2_) was used to quench the calcein fluorescence outside of the mitochondrial matrix. One-way ANOVA was performed, n=4-5 each group, p<0.0001 with significant comparisons * versus *Speg^+/+^,* † versus *Speg^-/-^* CMs untreated with CoCL_2_*.* **b**) Representative images of co-staining for Flur-4 (green) and MitoTracker (red). The bar graph (right) shows the quantitation (MFI), presented as mean±SEM. n=4-5 each group, Unpaired t-test was performed, p=0.9765. For quantitation of MFI in **a** and **b**, Fiji color threshold was performed to select the mitochondria area labeled by MitoTracker. Fiji color histogram was used to measure green MFI labeled by either calcein AM or Flur-4. Scale bars represent 20μm. **c**) Total protein was extracted from *Speg*^+/+^ adult hearts. Representative blots of total protein immunoprecipitated (IP) without antibody (input), with normal rabbit IgG (IgG), and with rabbit anti-mouse Speg antibody (Speg). The membrane was immunoblotted with anti-Speg (top rows) and VDAC (left, bottom panel), NCLX (middle, bottom panel) and MUC (right, bottom panel).

**
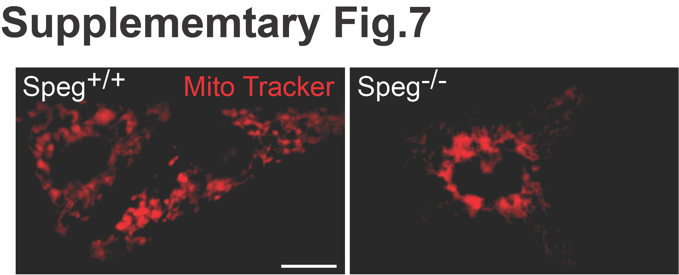
**

**Supplementary Figure 7.**  **Perinuclear distribution of mitochondria in *Speg*^-/-^ CMs.** CMs were harvested from E18.5 *Speg*^+/+^ (upper panel) and *Speg*^-/-^ (lower panel) hearts and stained for Mito tracker (red). Scale bar represents 10μm.

**
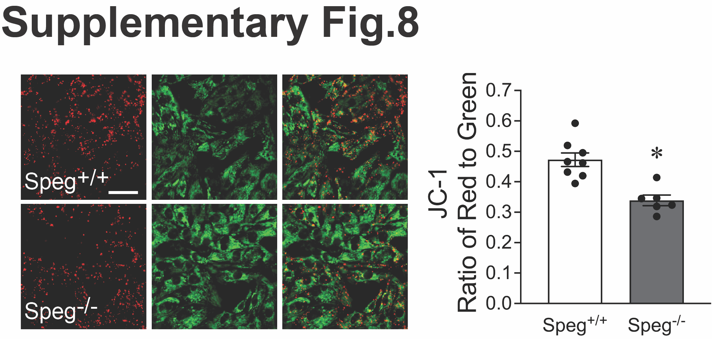
**

**Supplementary Figure 8. Abnormalities of mitochondrial membrane potential in E18.5 *Speg*^-/-^ hearts.** CMs were cultured in neonatal CM culture medium for 48 hours and then incubated with 2mΜ Tetraethylbenzimidazolylcarbocyanine iodide (JC-1) at 37 °C for 15 minutes. Confocal microscopy images were taken and analyzed by ImageJ.

Representative images of JC-1 staining in red (forming J aggregates) and green (depolarization, remaining as monomer) of *Speg*^+/+^ and *Speg*^-/-^ hearts. Quantitation of red to green ratio of JC-1 staining in bar graph, n=6-8 hearts for each group. The data are presented as mean±SEM. t-tests were performed, comparing *Speg*^+/+^ with *Speg*^-/-^ groups. * p=≤0.001 versus *Speg*^+/+^ CMs.


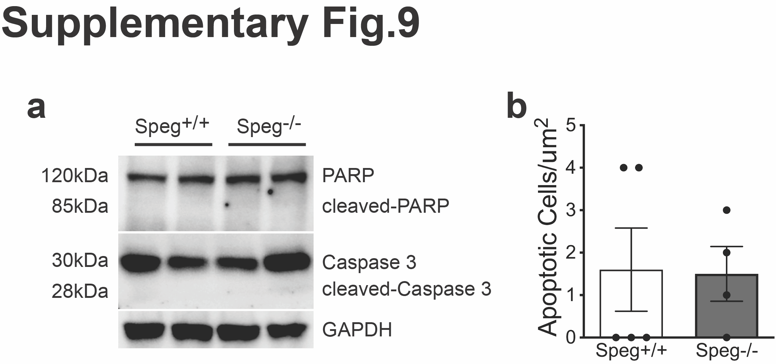


**Supplementary Figure 9. No increased apoptosis in *Speg*^-/-^ hearts.**

E18.5 hearts were harvested, and protein was isolated for western blotting. **a**) Representative Western blots for PARP/cleaved-PARP (top row), Caspase3/cleaved-Caspase3 (middle row), and GAPDH (bottom row), from *Speg*^+/+^ (left two lanes) and *Speg*^-/-^ (right two lanes). **b**) Quantitation of terminal deoxynucleotidyl transferase dUTP nick end labeling in *Speg*^+/+^ (white bar) and *Speg*^-/-^ (grey bar) hearts, n=4-5 for each group. The data are presented as mean±SEM apoptotic cells/µm^2^, Unpaired t-test was performed, p=0.9383.


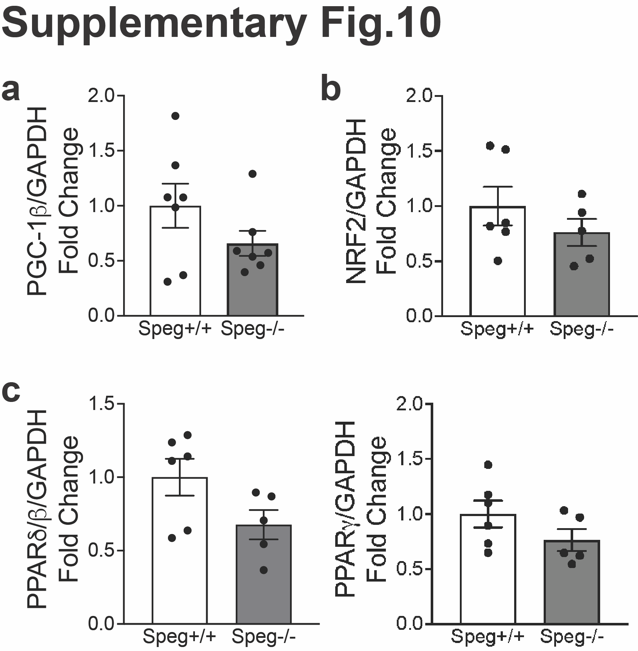


**Supplementary Figure 10. Expression of PGC-1β and downstream genes of PGC-1 in developing hearts.** Total RNA was extracted from E18.5 *Speg*^+/+^ (white bars) and *Speg*^-/-^ (grey bars) hearts. qRT-PCR assay for PGC-1β (**a**), NRF2 (**b**), PPARδ/β and PPARγ (**c**) was performed, n=5-7 for each group. The data are presented as mean±SEM, fold changed compared with *Speg*^+/+^ hearts, Unpaired t-tests were performed, p≥0.0824.


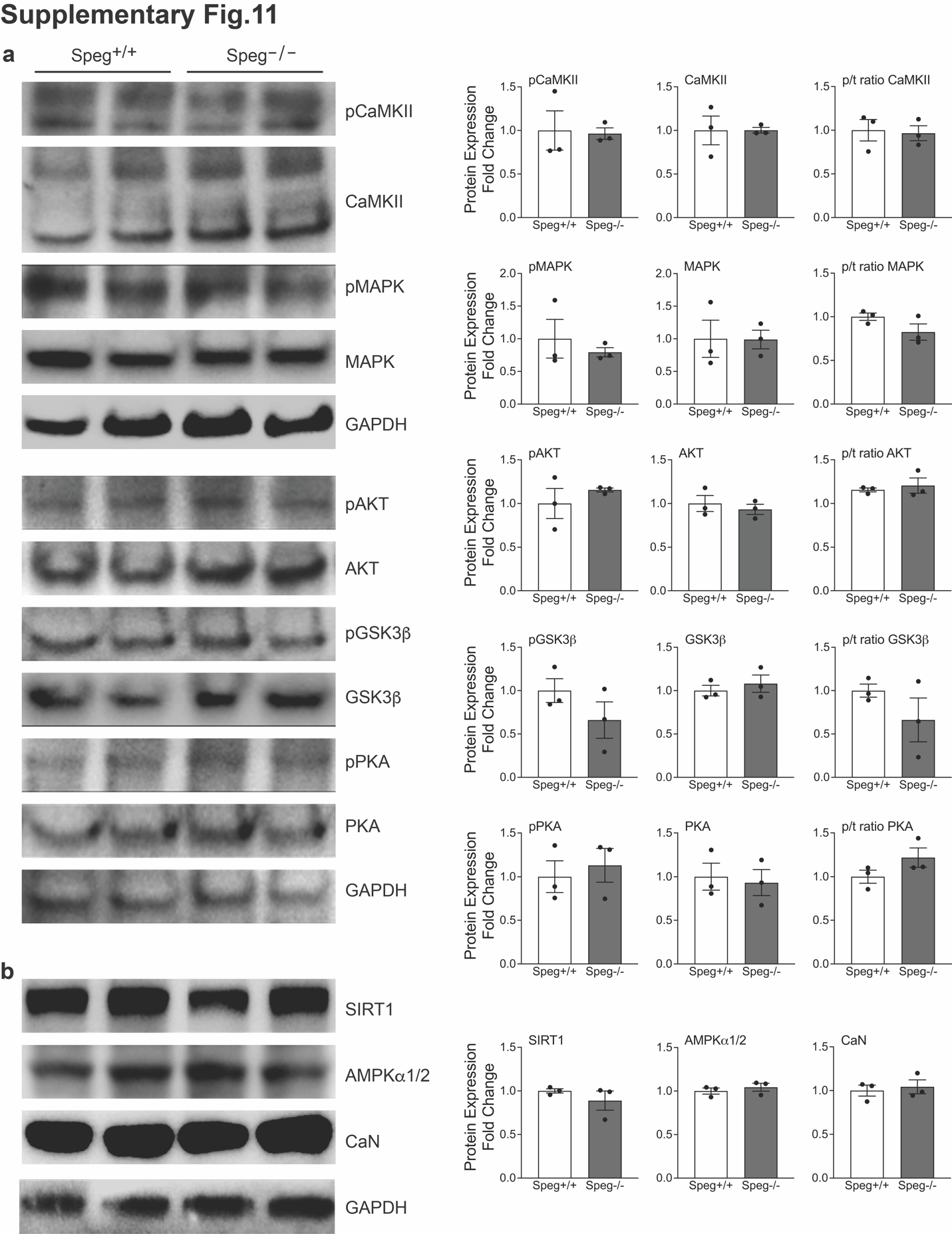


**Supplementary Figure 11. Expression of proteins upstream of PGC-1α in developing hearts.** Total protein was extracted from E18.5 *Speg*^+/+^ (white bars) and *Speg*^-/-^ (grey bars) hearts. Western blots (WBs) were performed, and antibodies used for blotting are shown in Supplemental Table 2. **a**) Representatives (left) and quantitation (right) of WBs, blotting with total- and phospho-antibodies. **b**) Representatives (left) and quantitation (right) of WBs, blotting with antibodies. n=3 for each group in **a** and **b**. The data are presented as mean±SEM, and Unpaired t-tests were performed. No differences in expression between any of the proteins, p≥0.1619.


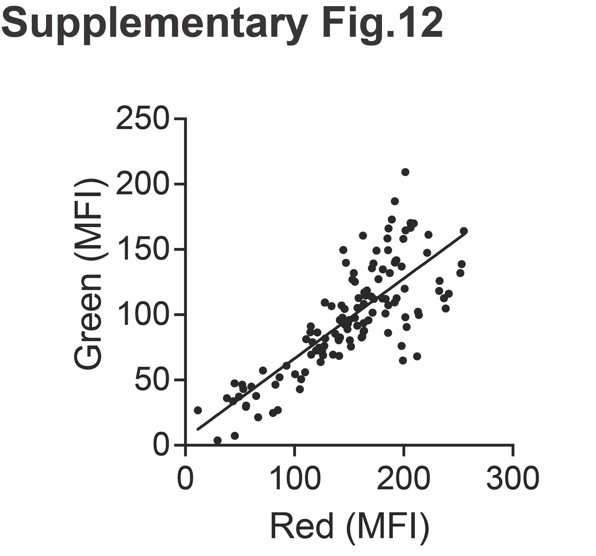


**Supplementary Figure 12. Colocalization of Speg and pPGC-1a expression between Z-line of CM.** E18.5 hearts were immunofluorescent staining for Speg (green, shown in Fig.5) and pPGC-1α (red, shown in Fig.5). Green and red mean fluorescent intensity (MFI) of confocal merged images were measured by Fiji color histogram. The yellow spots (co-staining area) in the area between z-lines was selected for measurement. R^2^=0.6121, p<0.0001.
